# Optimal dosage of group-based organized physical activity for enhancing social abilities in autistic children: insights from a multilevel meta-analysis

**DOI:** 10.1186/s12966-025-01787-8

**Published:** 2025-07-01

**Authors:** Jinrong He, Yikang Gong, Mingyue Yin, Lei Zhang, Xueping Wu

**Affiliations:** 1 School of Physical Education, Shanghai University of Sport, Shanghai, 200438 China; 2 School of Athletic Performance, Shanghai University of Sport, Shanghai, 200438 China

**Keywords:** Group-based organized physical activity, Social abilities, Autistic children, Meta-analysis

## Abstract

**Background:**

In response to current research trends emphasizing training programs to develop daily living skills in autistic children, this study employs a meta-analysis to explore the impact of group-based organized physical activity (GBOPA) on the social abilities of autistic children from multiple perspectives and further investigates its dose‒response relationship to define the “optimal” dose.

**Methods:**

We searched the PubMed, Web of Science, Embase, and Cochrane Library databases to identify relevant studies and screen their references. The effect size was calculated via Hedges’ g, and three-level random effects models were constructed via the *metafor* package in R. Moderation and regression analyses were conducted to explore significant influencing factors.

**Results:**

This study included 24 articles from ten countries and included 1,042 participants (aged 4.56–11.11 years). The meta-analysis results clearly show that GBOPA can significantly improve social abilities (g = 0.48, Q = 114.84), including social functioning (g = 0.50, Q = 62.97) and communication (g = 0.37, Q = 48.07), in autistic children. Moderation analysis indicated that different age groups and training frequencies significantly affected social ability (between-group difference: *p* < 0.05). Specifically, interventions for early childhood children (g = 0.65) and a frequency of five sessions per week (g = 0.69) significantly enhanced the training effects on social ability. The multivariate meta-regression analysis results suggest that the optimal intervention for improving social ability in autistic children consists of 40 training sessions, each lasting 50 min.

**Conclusions:**

GBOPA can improve the social abilities of autistic children, including social functioning and communication. On the basis of existing evidence, GBOPA should be prioritized for early childhood autistic children (5 sessions per week, 50 min per session), followed by a transition to a maintenance intervention strategy (1–2 sessions per week) after completing the 8-week foundational cycle (a total of 2,000 min of exercise).

**Supplementary Information:**

The online version contains supplementary material available at 10.1186/s12966-025-01787-8.

## Background

Social impairment is one of the two major core clinical features in children with autism spectrum disorder (ASD) [1], presenting clear developmental specificity during various stages of growth, including infancy (reduced eye contact) [2], preschool years (delayed social responses) [3], school age (non-verbal communication difficulties) [4], and adolescence (difficulty in understanding emotions) [5], which gradually emerge as neurodevelopment progresses [6]. Additionally, children are required to utilize a combination of motor skills in daily social activities, including gross motor abilities, such as running and jumping, as well as fine motor abilities, such as hand grip and object manipulation. While motor impairments are not a core diagnostic criterion for autism due to comorbid developmental disorders [7], two recent studies based on the SPARK large dataset indicate that the incidence of motor impairments in school-age children with ASD is as high as 86.9-88% [8], with a relative risk 22.2 times higher than that of typically developing children [9], specifically manifesting as bilateral coordination difficulties [10], slow movement speed [11], abnormal gait [12], and fine motor control deficits [13]. The “embodiment” theory offers a novel perspective for understanding the core social impairments in autistic children [9, 14]. This theory posits that human cognition, thinking, and emotions arise from the interaction between the body and the external environment [15, 16], emphasizing that movement is a crucial means of perceiving the social environment and plays a significant role in information processing [17, 18]. In recent years, increasing evidence has supported a connection between motor skills and social abilities in autistic children and typically developing children. Well-coordinated motor skills not only enhance the perception of external environmental cues but also promote the development of adaptive behaviors during interactions [7, 19, 20].

Therefore, given the correlation between motor skills and ASD, several reports call for further exploration of interventions that can help individuals with autism use motor skills more effectively in social interactions [21, 22]. Pellicano et al. also stated that autism research should prioritize the learning and development of daily living skills management [23]. As a result, community-based interventions that provide the most effective learning opportunities for autistic children in natural environments have garnered increasing attention from researchers in recent years [24]. A typical example is group-based organized physical activity (GBOPA), which involves two or more participants [25]. Considering the feasibility of previous interventions, Howells et al. (2019) defined GBOPA as a formal, organized physical activity training process with more than two participants [26, 27]. It is organized by clubs or recreational associations (including sports organizations, communities, schools, or educational organizations) under adult or coach supervision, with a child-to-coach/adult ratio greater than 1:1, and each session lasts no less than 30 min. This approach overcomes the limitations of previous physical activity interventions, which include only individual-based designs (e.g., jogging [28]). It creates more opportunities for autistic children to interact with peers, helps to better understand the impact of motor skills on children with ASD, and provides deeper insight into the influence of physical activity on social ability [29].

To date, only one meta-analysis has investigated the effect of the GBOPA on social ability (which encompasses social functioning and communication) in autistic children [26]. The analysis revealed that the intervention had a moderate effect on social functioning (g = 0.45, *p* < 0.001). While the findings are promising, the study also revealed that the GBOPA had a non-significant effect on communication (g = 0.13, *P* > 0.05), which contradicts recent results from other forms of GBOPA interventions aimed at improving communication in autistic children [30, 31]. This inconsistency may arise from significant differences in intervention parameters (such as duration, organization, and methods) across studies. Furthermore, the study reflects the limitations of the existing literature at the time (including only 4 studies), which did not account for any potential moderating factors or publication bias. It also overlooks the dependency between effect sizes (choosing one from multiple indicators), which results in reduced statistical power and limits the scope of the research topic [32]. In conclusion, the impact of GBOPA on the social abilities of autistic children remains unclear. Previous studies have had insufficient statistical power due to small sample sizes and methodological flaws, and significant differences in intervention parameters across studies have led to inconsistent conclusions. Additionally, the lack of exploration of factors such as GBOPA dosage and other exercise prescription variables has further limited its clinical applicability. Therefore, there is an urgent need for high-quality meta-analyses to fill the gaps in existing research, provide evidence-based guidance for clinicians, and ultimately develop more targeted and effective intervention programs for autistic children.

This study integrates potential moderating factors through three-level meta-analysis, quantifies the impact of the GBOPA on the social abilities of autistic children, and focuses on the “optimal dose” conceptual framework, offering scientific value in three key areas: (1) providing evidence-based guidance to clinicians for developing adaptive exercise prescriptions; (2) establishing a theoretical foundation for investigating the neurobiological mechanisms of the dose‒response relationship; and (3) offering quantitative support for family rehabilitation decision-making.

## Methods

This review was guided by the Preferred Reporting Items for Systematic Reviews and Meta-Analyses Statement (PRISMA) [34]. The review protocol was prospectively registered in PROSPERO and submitted online in August 2024 (ID: CRD42024609228). To align with the open science trend and enhance the transparency and reproducibility of the article, the relevant code and data have been uploaded to OSF (Open Science Framework: https://osf.io/t9aj4).

### Information sources and search strategies

Two researchers (JH and GY) independently analyzed the screening processes and results of the studies included in this review. The following databases were searched until August 2024: PubMed, Web of Science, Embase, and the Cochrane Library. Additionally, we searched ClinicalTrials.gov and the International Clinical Trials Registry Platform; detailed Boolean strings are provided in the Supplementary Material (Table S1). Given the potential timeliness issues related to the time effect, this study conducted a secondary literature search to ensure comprehensive coverage of the original studies (August 2024 to February 2025).

### Selection criteria

All the documents were screened and assessed according to the PICOS principle to determine their inclusion or exclusion criteria (a more detailed explanation of the inclusion criteria is shown in Supplementary Material 1.2).

1. Participants: All participants (3–12 years old) were diagnosed with autism spectrum disorder (ASD).
2. Intervention: The study included at least one intervention aimed at solely training the GBOPA.
3. Control group: Control groups in the included studies were not exposed to organized physical activity (OPA) programs.
4. Outcome: The outcome indicator included in this study was social ability.
5. Study design: This review included only experimental studies with pre- and post-test assessments and only RCTs and quasi-experimental studies.
6. The studies were written in English.
7. Studies were published in peer-reviewed journals.
8. Studies reported sufficient information to compute effect size.
9. Studies involving human participants.

### Study selection and data processing

The literature search was conducted in two stages. In the first stage, two researchers (JH and YG) identified the search keywords and used Rayyan to screen the literature according to eligibility criteria [35]. Upon completion of the screening, any disagreements (less than 1%) were discussed, and a consensus was reached. In the second stage, a third researcher (XW) reviewed the full texts of the identified articles and, in collaboration with the second researcher (YG), determined whether any articles could be excluded and reached a consensus. Data extraction was independently performed by two researchers (JH and YG), who achieved a consistency of 94.85%. For any inconsistencies, a consensus was reached after discussion with a third researcher (XW). We extracted the study identification data (i.e., author and year of publication) and moderating variables, which were categorized into population, intervention, control, and outcome variables on the basis of the PICO framework of evidence-based medicine [36]. Table 1 presents the extraction criteria for the moderating variables (a more detailed rationale is provided in Supplementary Material 1.3).

**Table 1:** Standardized protocol used for data extraction from the selected studies

PICO	Moderator	Category
Population	Age	Early childhood (3-6years old); Early-school-age (6-8.5years old); Pre-adolescence(8.6-12years old)
Intervention	Training task	Motor skill-based interventions; Motor technology-based interventions
Intervention	Training frequency	Once per week; Twice per week; Three times per week; Four times per week; Five times per week
Intervention	The time point of the test	Post-tests (tests within seven days); Follow-up (more than 30 days from the last training).
Intervention	Group size	Small (less than or equal to 3 people); Large (greater than 3 people)
Control	The control group	Active control group (additional non-OPA and non-social skills programs with the same training strategy as the experimental group); Passive control group (No additional intervention and only participated in the pre-test and post-test).
Outcome	The type of social abilities	**Social abilities**: Social function; Communication

### Statistical analysis

Given that many studies report multiple effect sizes, violating the assumption of effect size independence, this study employed a three-level meta-analysis to account for dependencies between effect sizes and retain as many effect sizes as possible [33]. Hedges’ g effect size (g) was employed in the data analysis, following established guidelines and prior research [37, 38, 39, 40]. A positive effect size indicates that the experimental group achieved greater gains in social ability than did the control group. The effect sizes and variances were calculated in Excel, according to the methodology outlined by Aksayli et al. [41]. The metafor package in R version 4.4.0 (R Core Team, 2024) was used for subsequent statistical analyses [42]. Detailed formulas for calculating the effect sizes and comprehensive statistical procedures are provided in the supplementary material (1.4 Statistical Analysis).

### Risk of bias and quality of evidence

The RoB2 (Cochrane Risk of Bias Tool 2) evaluates the quality of included studies across five domains: bias due to the randomization process, bias from deviations from intended interventions, bias due to missing outcome data, bias in the measurement of outcomes, and bias from selective reporting of results [43]. We selected RoB2 because it assesses the risk of bias for individual outcomes rather than for the entire trial. Additionally, Sterne et al. recommended the use of RoB2 in meta-analyses to evaluate the overall risk of bias on the basis of the analysis of single outcomes [43]. Two researchers (JH and YG) independently assessed the risk of bias for the included studies, and any discrepancies were resolved through discussion with a third researcher (XW).

Furthermore, Carey et al. argued that evaluating the overall quality of evidence requires assessing the validity of individual study outcomes [44]. Consequently, the quality of evidence in this meta-analysis was assessed via the Grading of Recommendations, Assessment, Development, and Evaluation (GRADE) system, which consists of five criteria [45]: limitations, inconsistency, indirectness, imprecision, and publication bias. Each criterion was rated on a severity scale as follows: not serious (no downgrade), serious (downgrade by one level), and very serious (downgrade by two levels). The overall quality of evidence was categorized into four levels: high, moderate, low, and very low, and the results are presented in an evidence summary table.

## Results

**Fig. 1:**
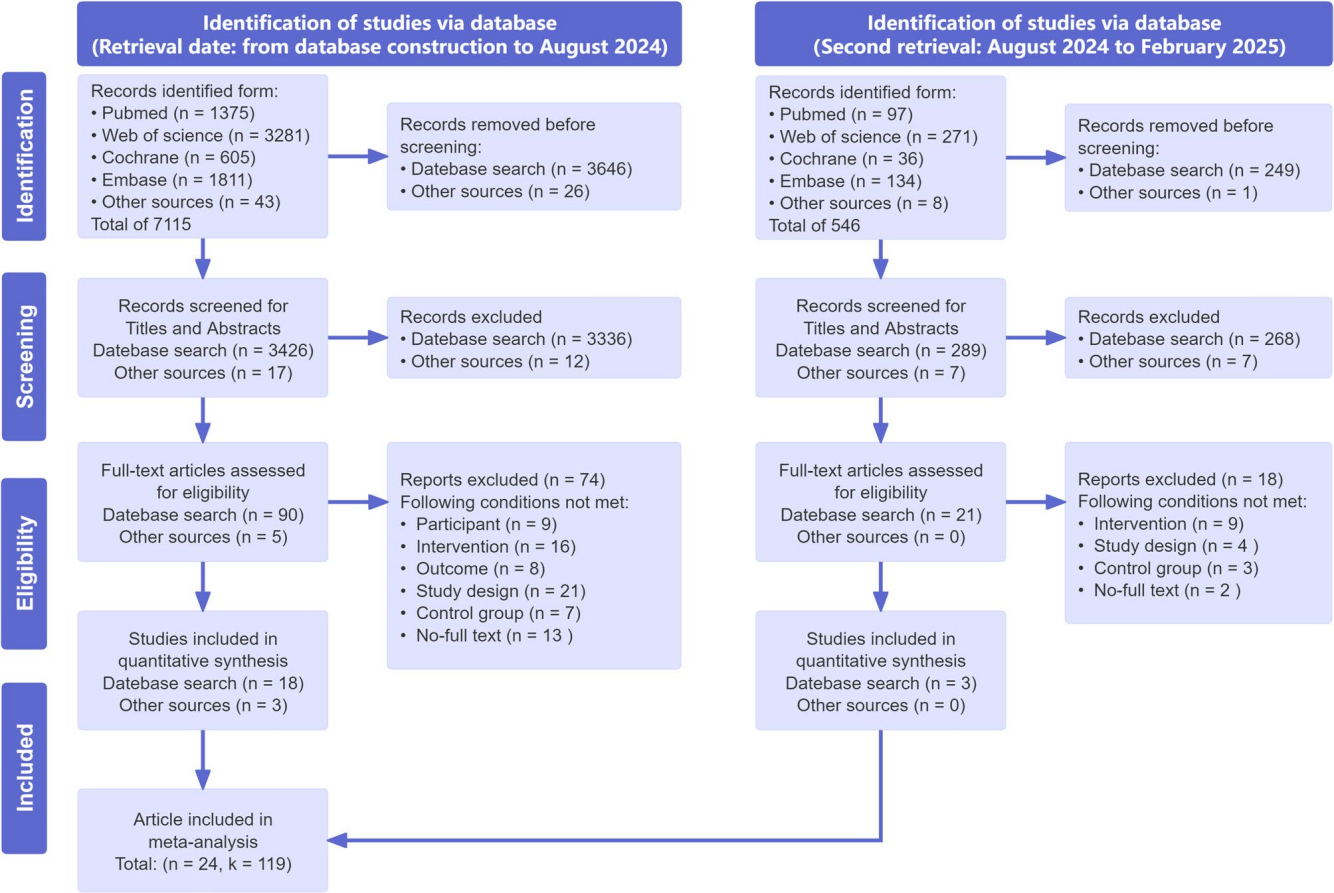
Flowchart of the selection and inclusion process, following the PRISMA Statement. Note: n represents the number of articles, and k represents the number of effect sizes

An initial search yielded 7,115 studies based on the inclusion criteria (from database construction to August 2024), whereas the second search (from August 2024 to February 2025) retrieved 546 studies. Of these, 3,922 duplicates were excluded. Following title and abstract screening, 116 articles were selected for full-text review, 24 of which met the inclusion criteria and were included for data extraction and evaluation (see Fig. 1). The total sample size of the included studies was 1042 children divided into two groups: the intervention group comprised 555 individuals (52%), whereas the control group consisted of 487 individuals (48%). The participants’ average age ranged from a minimum of 4.56 [46] years to a maximum of 11.11 [47] years. The years of publication of these studies varied from 2009 to 2024. Geographically, the selected studies represented multiple countries: six studies were conducted in China [46, 47, 48, 49, 50, 51], four in the United States [52, 53, 54, 55], four in Iran [56, 57, 58, 59], two in Australia [31, 60], two in Italy [61, 62], and two in Brazil [30, 63]; and one study was conducted in Spain [64], Greece [65], Turkey [66], and Israel [67]. (More detailed study characteristics are shown in Supplementary Material 2.1).

### The overall effect of GBOPA on social ability

To assess the overall effect size of GBOPA on social ability, we synthesized all effect sizes, disregarding the types of outcome measures and control groups (Fig. S1). The three-level random effects model indicated a significant overall effect size of g = 0.58, with a 95% confidence interval (CI) of [0.40, 0.75] and a 95% prediction interval (PI) of [− 0.22, 1.37] (*p* < 0.001). Similarly, the two-level random effects model revealed a significant overall effect size of g = 0.49, with a 95% CI of [0.42, 0.57] and 95% PI of [− 0.06, 1.05] (*p* < 0.001). Cochran’s Q statistic (Q) observed significant heterogeneity (Q_(118)_ = 235.66, *p* < 0.001).

### Sensitivity analysis

To investigate whether outliers could distort the results of the meta-analysis [68], we conducted a sensitivity analysis that identified five outliers (g = 2.93, g = 2.90, g = 2.30, g = 2.21, g = 2.24 [48, 49, 63]; see Fig. S2 and Table S3). After excluding outliers, the meta-analysis revealed that the three-level random effects model resulted in a slight reduction in the overall effect size, but it remained significant (g = 0.48, 95% CI [0.38, 0.59], 95% PI [0.12, 0.84], *p* < 0.001). Similarly, the overall effect size in the two-level random effects model also showed a decreasing trend, but it remained significant (g = 0.40, 95% CI [0.35, 0.45], 95% PI [0.30, 0.50], *p* < 0.001). In contrast, the heterogeneity was not significant (Q_(113)_ = 114.84, *p* = 0.434). Although the results indicated no significant difference in the average effect size before and after excluding outliers, the prediction interval, which accounts for between-study variability, significantly narrowed after excluding outliers, with both random effects models demonstrating an overall positive effect. Therefore, to ensure the robustness of the study and the consistency of the overall impact, outliers were excluded from subsequent analyses.

### Heterogeneity analysis

Structural equation modeling was used to investigate the sources of total variance [38]. The results revealed that the sampling variance (level 1) accounted for 73.39%, the within-study variance (level 2) accounted for 0.13%, and the between-study variance (level 3) accounted for 26.60%. The likelihood ratio test (LRT) revealed that the between-study variance (level 3) was significant (LRT = 9.89, *p* < 0.001, one-tailed), suggesting that the three-level model was superior to the two-level model (with level 3 set to zero). Conversely, the within-study variance (level 2) was not significant (LRT < 0.01, *p* > 0.49, one-tailed), indicating that the three-level model did not outperform the two-level model (with level 2 set to zero). Although the three-level model did not significantly outperform the two-level model when level two was set to zero, using the three-level model was meaningful because when studies reported multiple effect sizes in the meta-analysis, a nested pattern existed between effect sizes and studies (variance between studies at level three = 26.60%) [37]. In this case, the three-level model better represents the data generation process, illustrating how the data are “generated” [69]. Therefore, in the subsequent report, we present the results from the three-level random-effects model.

### Overall effect of the results for different comparison groups

**Fig. 2:**
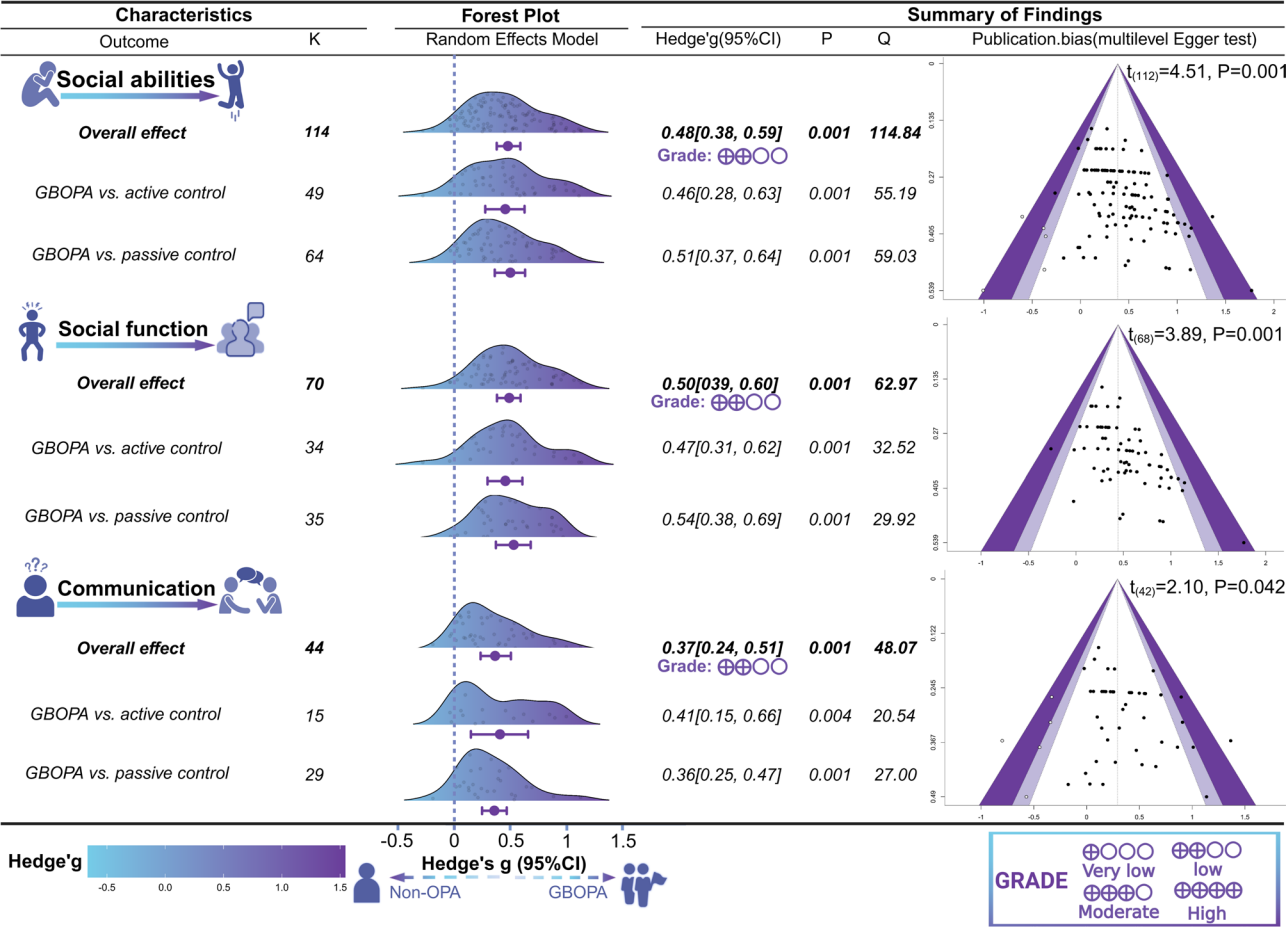
The results of the GBOPA on social ability and its sub-indicators in autistic children were compared with those of the active and passive control groups. Note: k represents the number of effect sizes; Q represents the Cochran’s Q statistic (all results were non-significant)

We also included the types of outcome measures and control groups to analyze the overall effect sizes of the active control group compared with those of the passive control group across different outcomes (see Fig. 2). The results revealed that, compared with the active control group, both overall social ability and the two dimensions based on the Diagnostic and Statistical Manual of Mental Disorder s- Fourth Edition (DSM-IV: social functioning and communication) had significant overall effect sizes (*p* = 0.001–0.004). Additionally, the passive control group presented greater effect sizes for social ability outcomes than did the active control group (∆g = 0.5).

### Moderator analysis

**Fig. 3:**
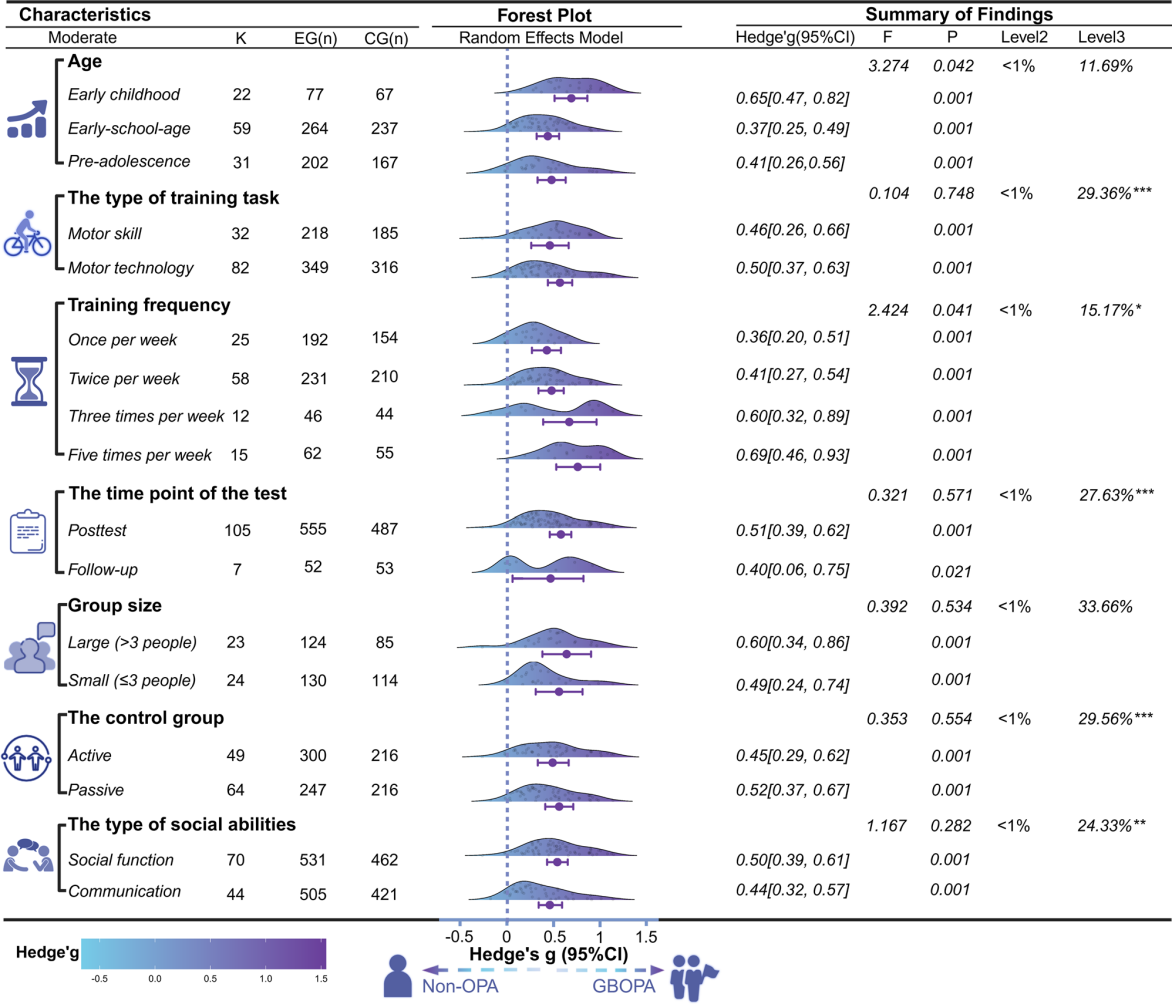
The results of moderators of the effect of GBOPA on social ability. Note: CI, confidence interval; PI, prediction interval; k represents the number of effect sizes; n represents the sample size; *p < 0.05, **p < 0.01, *** *p* < 0.001

We examine the effects of a series of moderating variables on the overall effect (see Fig. 3). Because the robust variance estimation (RVE) method requires at least five effect sizes to observe a combined effect [70], the groups with four training sessions per week in the training frequency moderator variable were excluded because of insufficient effect sizes. The results revealed that these two variables were statistically significant. First, the moderating effect of age was significant, as indicated by the F-statistic (F_(2,109)_ = 3.274, *p* = 0.042), with the largest effect size observed in early childhood children (g = 0.65, 95% CI [0.47, 0.82], *p* < 0.001). Pairwise comparisons indicated that the effect size in early childhood children was significantly greater than that in the other age groups (t_(109)_ = 2.51, *p* < 0.01). Second, the moderating effect of training frequency was significant, F_(4,109)_ = 2.424, *p* = 0.041, with the greatest effect size observed in the group with five training sessions per week (g = 0.69, 95% CI [0.46, 0.93], *p* < 0.1). Pairwise comparisons revealed that the effect size in the five-time-per-week group was significantly greater than that in the other frequency groups, t_(109)_ = 5.84, *p* < 0.01.

### Multivariate meta-regression

**Fig. 4:**
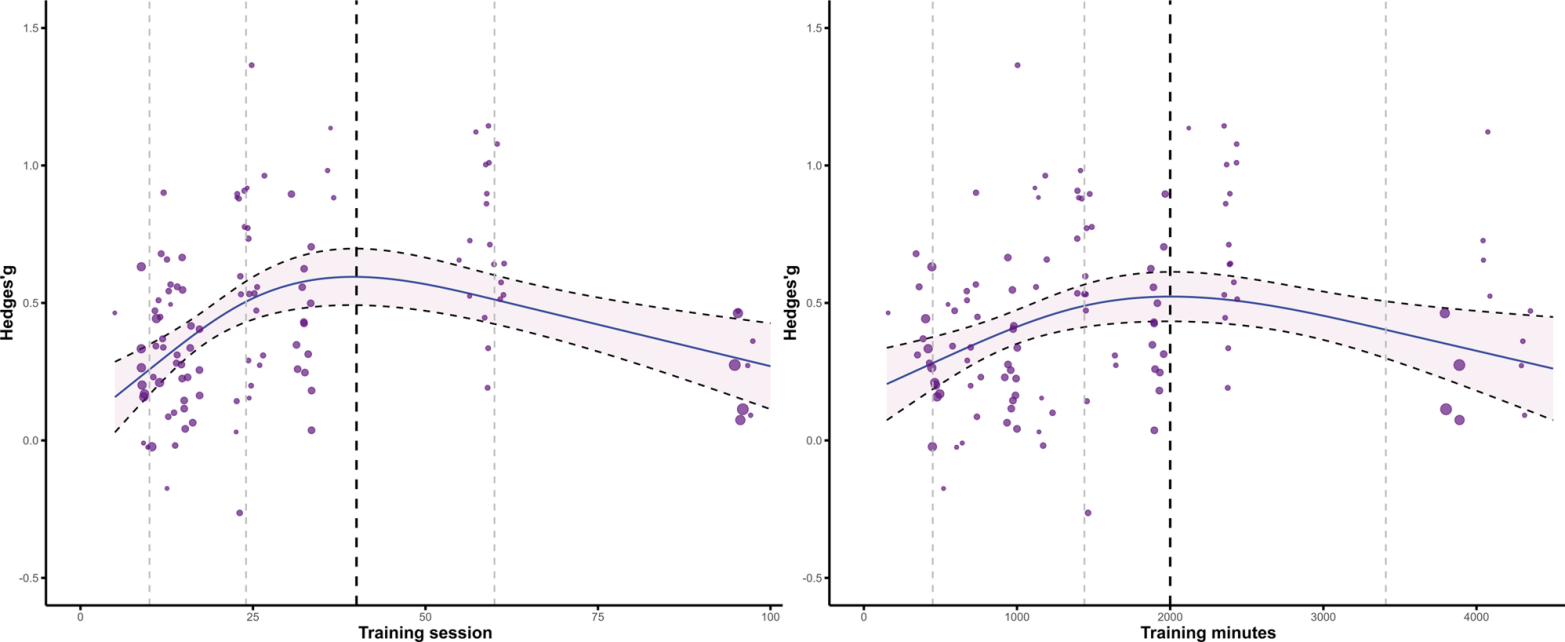
The cubic spline regression model captures the nonlinear relationship between intervention dosage (including both training sessions and duration) and the corresponding effect size. For the training sessions, the thin dashed vertical lines indicate the knot locations at 10, 24, and 60 sessions. For intervention duration, the knot locations are marked at 450, 1440, and 3408 min. The thick dashed vertical line highlights the threshold at which the intervention effect reaches its maximum. The shaded region illustrates the 95% confidence interval

To explore whether an “optimal dose” exists, we performed an exploratory multivariate meta-regression analysis to investigate the relationship between training dose (sessions and duration) and effect size and to examine potential thresholds (see Fig. 4). The results showed that the cubic model with three knots provided the best fit for the relationship between the intervention dose (training sessions and duration) and intervention effects. No significant Q-statistic for residual heterogeneity (QE) was found for the number of training sessions [QE _(df = 111)_ = 95.08, *p* = 0.859], and the moderator factor test for spline terms was significant for all spline coefficients [F _(2,111)_ = 9.87, *p* < 0.001], indicating that the heterogeneity of training sessions is not significant and has a significant impact on the intervention effect. The fitted curve analysis indicated that the GBOPA intervention program for autistic children peaked after 40 sessions. Similarly, no significant residual heterogeneity was found in the training duration [QE _(df = 111)_ = 103.20, *p* = 0.688], and the moderator factor test for spline terms was significant [F _(2,111)_ = 5.82, *p* = 0.004], indicating that the heterogeneity of training duration is not significant and has a significant impact on the intervention effect. The fitted curve analysis revealed that the total duration of the GBOPA intervention for autistic children peaked at 2000 min. In conclusion, the optimal intervention dose for improving social ability in autistic children consisted of 40 training sessions, each lasting 50 min.

### Risk of bias

We used RoB2 to assess the quality of the included studies. Among the 119 effect sizes, four from one study were rated as low risk (4/119, 3.36%) [63], 108 from 20 studies were rated as having some concerns (108/119, 90.75%), and seven from three studies were rated as high risk (7/119, 5.88%; see Fig. S3 and Fig. S4) [30, 53, 64]. Notably, most studies were rated as “having some concerns” for two reasons. First, almost all participants were recruited voluntarily through community or hospital channels, which violated the concealment standard for participant allocation in the randomization process. Specifically, according to RoB2, if the randomization process does not follow the standard protocol, there could be an issue of exaggerating treatment effects, which could lead to “some concerns” or even “high risk” in the domain of “bias in selection of the reported result”, affecting the overall evaluation. Second, many previous clinical studies involving special children have not been conducted. Specifically, according to RoB2, if a study is not preregistered, it becomes difficult to determine whether the reported results align with the planned protocol, leading to a judgment of “some concerns” in the “bias in selection of reported results” domain, which affects the overall evaluation. Therefore, studies rated as having “some concerns” do not necessarily indicate low quality, which may be due to the particularity and scarcity of the selection process for autistic children and the pre-registration of adapted physical activity in clinical medicine [71].

### Publication bias

We assessed the impact of publication bias in small studies via funnel plots [72], Egger’s regression test [73], and the trim-and-fill method recommended by Duval and Tweedie [74]. The funnel plot displayed asymmetry (Fig. 2), indicating a potential risk of publication bias. Egger’s regression test of the two-level model revealed a high risk of publication bias (t_(112)_ = 5.03, *P* = 0.001). The results of the multilevel Egger test, which incorporated modified predictive factors, were also significant (t_(112)_ = 4.51, *P* = 0.001). The trim-and-fill with “R0” showed that 5 effect sizes were missing to the left of the mean effect size. After correction, the overall effect size was significant (g = 0.38, 95% CI [0.33, 0.44]; *p* < 0.001). This was not significantly different from the observed effect size (g = 0.48, 95% CI [0.38, 0.59], *p* < 0.001). On the basis of the above results, although there may be a high risk of publication bias in the extraction analysis, this did not significantly affect the results.

### Level of evidence quality assessment

We employed the GRADE system to evaluate the quality of evidence (Table S4) [45]. In addition to assessing the two primary outcome measures of social abilities and optimal dose, we included evidence ratings for two moderating variables related to social abilities: social functioning and communication. However, the quality of evidence was downgraded owing to several issues, including unclear allocation strategies in some studies, study imprecision, and low-level publication bias. Consequently, the overall assessment results were as follows: (1) social ability outcome measure: low quality of evidence, (2) categorical variable social functioning: low quality of evidence, and (3) categorical variable communication: low quality of evidence. (4) Training session optimal dose: low quality of evidence; and (5) Training duration optimal dose: low quality of evidence.

## Discussion

**Fig. 5:**
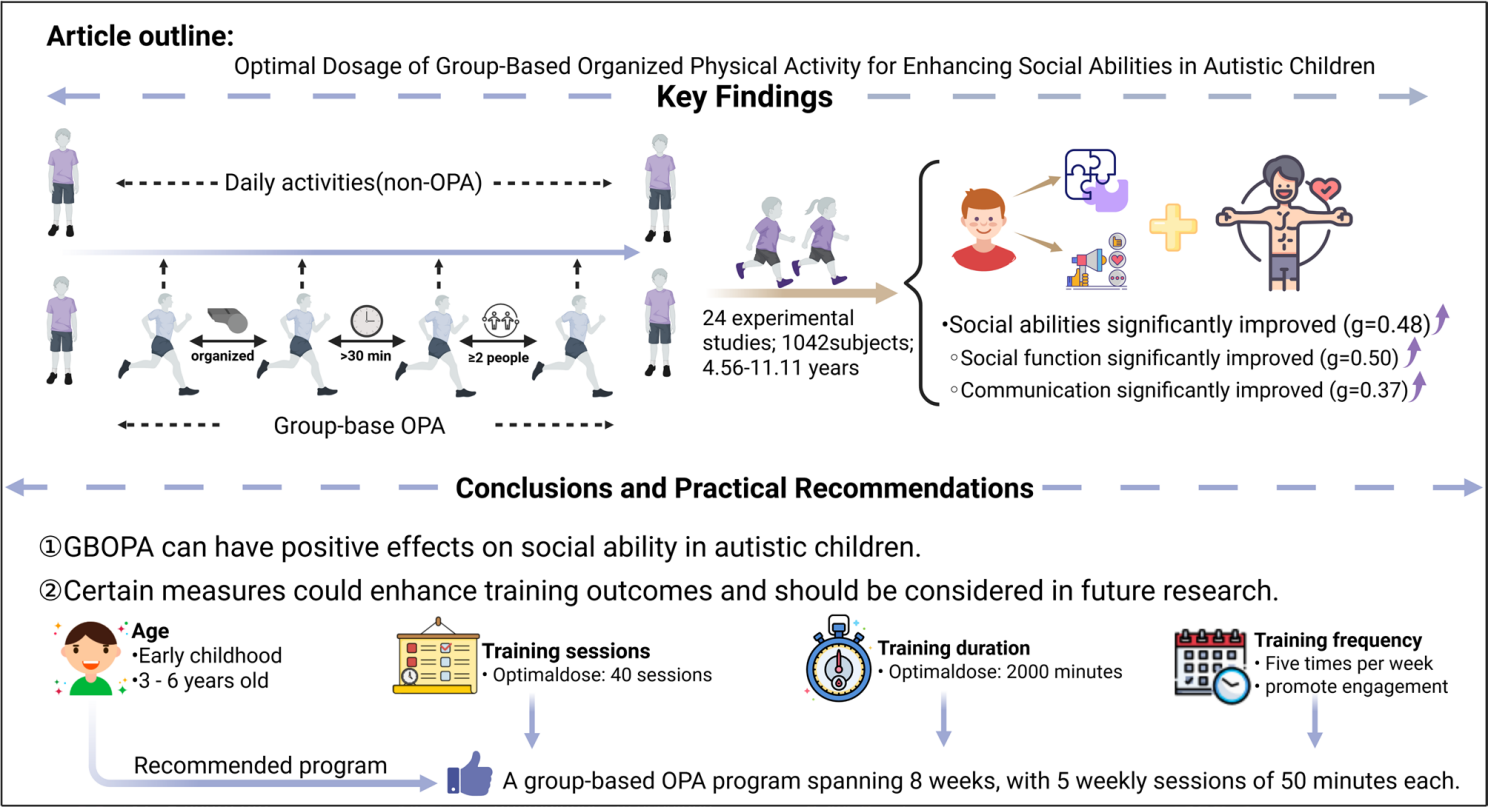
The outline of the article

This article systematically reviews 24 experimental studies (*N* = 1042) assessing the effects of GBOPA on social ability in autistic children and explores the potential moderating effects of variables such as participant characteristics and intervention protocols (see Fig. 5). The meta-analysis results are clear: GBOPA can significantly improve social abilities, including social functioning and communication, in autistic children. The moderation analysis indicates that interventions for early childhood children, combined with a frequency of five sessions per week, can significantly improve the training effects. Furthermore, according to the currently available literature, the optimal intervention dos*e* for improving social ability in autistic children is 40 training sessions, each lasting 50 min.

### Effect of GBOPA on social ability

GBOPA can significantly improve the social abilities of autistic children. First, GBOPA had a statistically significant effect on the social abilities of autistic children, even in the active control group. Second, the overall heterogeneity was low and non-significant. Third, the overall effect size of the passive control group was greater than that of the active control group, thus supporting our hypothesis. Fourth, while only 14 of the 49 effect sizes for social abilities in autistic children showed fully significant confidence intervals compared with the active control group [30, 46, 48, 50, 51, 55, 57, 60, 62], several confidence intervals approached significance [30, 46, 48, 50, 51, 55, 57, 60, 62]. Additionally, the original studies reported a consistent pattern: autistic children who participated in GBOPA interventions showed an improvement in social ability, whereas the control group remained unchanged [30, 46, 48, 50, 51, 55, 57, 60, 62]. Among the non-significant effect sizes, some studies indicated that while overall social abilities were not significant, more detailed functional tests, such as social cognition [55], social participation [48], communication [48], and social motivation [62], revealed the beneficial effects of GBOPA. Therefore, this study supports the idea that GBOPA can significantly improve the social abilities of autistic children. This finding aligns with the theoretical hypothesis that group exercise interventions facilitate the acquisition of social skills through role collaboration and multimodal social scenarios [75]. Research indicates that peer interaction and the enjoyable atmosphere of group activities during exercise can engage and refine the prefrontal cortex of individuals with autism, as well as other brain regions involved in large networks (such as the Default Mode Network and cortical-limbic system) [76]. This, in turn, improves abilities such as emotional regulation and social cognition and enhances problem-solving skills in social participation [76].

With respect to social functioning, we found that, compared with the active control group, GBOPA also had a statistically significant impact. Meta-analysis enhances statistical power by synthesizing multiple studies, enabling the detection of true intervention effects [77]. Therefore, the results of this study support the view that the GBOPA can improve the social functioning of autistic children. This finding is consistent with previous research, which suggests that improvements in social functioning primarily result from the dual effects of internalizing social rules and reinforcing cooperative behavior [78]. In GBOPA, participants clarify their roles (e.g., forward, goalkeeper), learn to take turns, wait, and share resources (e.g., passing, defending) through dynamic interaction. This process requires children with ASD to adapt to others’ behaviors within the “cooperation-competition” framework, thereby activating the mirror neuron system and enhancing their perception and imitation of social cues [79]. Moreover, previous meta-analyses have been inconclusive regarding whether GBOPA can improve communication in autistic children [26, 80]. In this study, GBOPA significantly improved communication compared with the active control group, with relatively low and non-significant heterogeneity, which supports our hypothesis. This result is consistent with recent studies showing that exercise prevents abnormal SHANK family protein abundance in individuals with autism, thereby improving both language and non-language abilities and alleviating core autism symptoms [81, 82, 83, 84]. Therefore, participation in various GBOPA activities can significantly increase communication in autistic children, which has clinical implications, as improved communication can reduce the risk of internalizing symptoms (such as anxiety or depression) and enhance social participation and future employment opportunities [85, 86].

### The moderator

Through moderation effect analysis, we found that the training effect of the GBOPA can be strengthened under certain conditions, providing valuable insights for future practice and research. Training frequency was a significant moderating variable, with five sessions per week showing the greatest effect size. This finding is consistent with previous research, which suggests that interventions targeting specific behavioral development in autistic children require intensive and frequent training to achieve significant effects [87, 88]. Additionally, GBOPA maximizes the participation of autistic children by providing structured social interaction opportunities and offering personalized activity plans during the intervention while fostering reciprocal friendships [89]. Therefore, the GBOPA intervention, with five sessions per week, may yield more favorable outcomes in the social abilities of autistic children.

Second, as hypothesized, different age groups were key moderating variables influencing the impact of GBOPA on social ability, with early childhood showing the greatest training effect. From a neurodevelopmental perspective, this may be due to the plasticity window of the prefrontal–limbic system neural circuits during this stage [90, 91]. During this period, synaptic pruning is not yet complete, and the high sensitivity of the mirror neuron system to action observation and imitation allows role-playing group activities to effectively promote functional integration of the social brain network through action synchronization mechanisms [79, 92, 93]. Additionally, the effect sizes significantly decreased during preschool (6–8.5 years) and early adolescence (8.6–12 years). This may be closely related to the stage transitions emphasized in Erikson’s theory of psychosocial development; as age increases, social interaction evolves from basic rule acquisition to adaptation to more complex contexts [94]. However, if current intervention programs continue to use standardized training processes without incorporating peer relationship building (such as group identity formation) and lack targeted designs for emotional empathy development, they may hinder the transfer of higher-level social skills from structured scenarios to real-life social situations [95]. Furthermore, a study observing “joint action” at different age stages in autistic children revealed that the primary phase of improvement in motor coordination and social function occurs in early childhood and stabilizes in early adolescence [95]. Therefore, the use of GBOPA interventions in early childhood may yield more favorable results.

Dose‒response curve analysis revealed that GBOPA improvement in social ability in children with ASD exhibited nonlinear characteristics and a clear threshold effect. This result supports the findings of the meta-analysis by Sandbank et al., who reported no significant positive correlation between intervention dose and effect size [70]. The analysis revealed that when the intervention dose reached 40 training sessions (total duration of 2000 min), the effect size peaked. After two significant moderating variables are combined, the findings of the present study also confirm the “early intensive strategy” in the field of behavioral intervention [96, 97, 98]. This strategy involves providing intensive stimulation during the early stages of intervention (five times per week for eight weeks) for early childhood children to establish stable neuro-behavioral connections, thereby laying the foundation for later skill generalization [96, 97, 98]. Additionally, from a neurodevelopmental perspective, intensive training accelerates long-term potentiation of synaptic efficacy through repeated, coordinated activation of the prefrontal‒limbic system, promoting functional reorganization in socially relevant brain regions (e.g., the superior temporal sulcus and anterior cingulate cortex) [99, 100]. Notably, the dose‒response curve showed a significant plateau after 40 sessions, which may have resulted from repetitive social stimuli diminishing the effectiveness of behavioral reinforcement [101]. Furthermore, excessive engagement in structured activities may cause children with ASD to reach their biological limits of cognitive load tolerance, triggering negative emotional rebounds, such as anxiety [102, 103, 104]. This nonlinear relationship offers dual insights for clinical practice. First, during GBOPA intervention, it is essential to create new social environments and opportunities to avoid repetitive social stimuli. Second, after the threshold is reached, the intervention should transition to a maintenance phase (e.g., 1–2 times per week), and intermittent reinforcement should be used to consolidate the benefits of neural plasticity. These findings provide quantitative evidence to address the “dose ambiguity” dilemma in non-pharmacological interventions, and caution that blindly extending the intervention period may violate the principles of neural metabolic homeostasis [105].

### Study limitations and implications

To the best of our knowledge, this is the first review to incorporate multiple moderating variables and explore the intervention dose threshold of GBOPA on social abilities in autistic children, thus providing valuable insights for future practical applications. However, the present study should be interpreted with several limitations. First, the number of studies and sample sizes within the studies was small, and publication bias was present. Although this did not significantly affect the results, it may have reduced the statistical power to some extent. Second, since many original studies did not stratify children by autism severity [48, 59, 60, 106] or sex [30, 55, 56] when implementing interventions or reporting results, this study compared only autistic children across different age groups. However, autism is a complex, heterogeneous disorder with diverse etiologies, subtypes, and developmental pathways [107, 108]. Therefore, future empirical studies should compare the effectiveness of GBOPA across autistic children who differ in genetic variations, comorbidities, and sex.

## Conclusions

GBOPA can improve the social abilities of autistic children, including social functioning and communication. On the basis of existing evidence, this study recommends prioritizing intensive GBOPA (5 sessions per week, 50 min per session) for early childhood autistic children, followed by a transition to a maintenance intervention strategy (1–2 sessions per week) after completing the 8-week foundational cycle (totaling 2400 min of exercise) to optimize the dose‒effect relationship for neurobehavioral improvement. While this meta-analysis synthesizes existing research, it is limited by the available data and the quality of the original studies and cannot substitute for large-scale empirical research. Therefore, future studies should include extensive randomized controlled trials to validate these findings.

## Electronic supplementary material

Below is the link to the electronic supplementary material.

Supplementary Material 1

## Data Availability

The data and code supporting this study were uploaded to Open Science Framework (https://osf.io/t9aj4), and the protocol for the review was prospectively registered in PROSPERO and submitted online in August 2024(ID: CRD 42024609228).

## Abbreviations

GBOPA: Group-Based Organized Physical Activity
ASD: Autism Spectrum Disorder
SPARK: Simons Foundation Powering Autism Research for Knowledge
PRISMA: Preferred Reporting Items for Systematic Reviews and Meta-Analyses Statement
PROSPERO: International Prospective Register of Systematic Reviews
PICOS: Population, Intervention, Comparison, Outcomes and Study
OPA: Organized Physical Activity
RCT: Randomised Controlled Trial
RoB2: Cochrane Risk of Bias Tool 2
g: Hedges’ g
GRADE: Grading of Recommendations, Assessment, Development, and Evaluations
CI: Confidence Interval
PI: Prediction Interval
LRT: Likelihood Ratio Test
RVE: Robust Variance Estimation
Q: Cochran’s Q statistic
F: F-statistic
QE: Q-statistic for Residual Heterogeneity
DSM-IV: Diagnostic and Statistical Manual of Mental Disorder s- Fourth Edition
SHANK: SH3 and Multiple Ankyrin Repeat Domains
