# Supplementary material for: Optimal dosage of group-based organized physical activity for enhancing social abilities in autistic children: insights from a multilevel meta-analysis
Source: Int J Behav Nutr Phys Act. 2025 Jul 1;22:87. doi: 10.1186/s12966-025-01787-8 (PMC12210644; doi:10.1186/s12966-025-01787-8)
Supplement: Supplementary file 1 — Supplementary Material 1 [file 12966_2025_1787_MOESM1_ESM.docx]

**Supplementary material** for

**Optimal Dosage of Group-Based Organized Physical Activity for Enhancing Social Abilities in Autistic Children: Insights from a Multilevel Meta-Analysis**

# 1 Supplementary Method

## 1.1 Search Strategy

**Table S1** Complete search strategy for each database.

| 1. organized | TI/AB:“extra-curricular” OR Communit* Club* OR Structure* OR Intervention* “community-based” OR organiz* OR organise* OR Communit* |
| --- | --- |
| 2.Autism Spectrum Disorder | Mesh: "Autistic Disorder" OR "Asperger Syndrome" OR "Autism Spectrum Disorder" OR "Rett Syndrome"  TI/AB: ASD OR Autis* OR "Autism Spectrum Disorder" OR Asperger* OR "Asperger* syndrome" OR "Pervasive developmental disorder*" OR PDD OR PDDNOS OR "Rett Syndrome" OR "Rett* disorder" OR "Kanner* Syndrome" OR “Neurodevelopmental disorders” |
| 3 Physical activity | Mesh: "Youth Sports" OR "Sports for Persons with Disabilities" OR Sports OR Exercise  TI/AB:Sport* OR Exercis* OR Train* OR Program* OR “Locomotor Activit*” OR “Physical* therap*” OR “physical* activit*” OR “motor activit*” OR “Physical* Exercis*” OR Soccer OR Swim* OR Aquatic* OR Dive OR Diving OR Football OR Basketball OR Cricket OR Tennis OR Rugby OR Danc* OR Athletic* OR “Martial art*” OR Netball OR Hockey OR Gym* OR “horse rid* OR “horseback rid*” OR Equestrian OR Baseball OR Judo OR Cycling OR Surf* |
| 4.Population | Mesh: Child OR "Child, Preschool"  TI/AB:Child* OR "School-age*" OR "young child*" OR Kid* OR Boy* OR Girl* OR Male* OR Female* OR "Primary-age*" OR Preschool OR “3-12 year” |
| 5.1 AND 2 AND 3 AND 4 | |

## **1.2 Selection Criteria**

The studies were included according to the following criteria:

(1)Participants: All participants were diagnosed with neurodevelopmental disorders according to ICD-11 (World Health Organization, 2022) and met the behavioral indicators specified in the ICD-11^[1]^. Alternatively, participants were diagnosed with autism spectrum disorder (ASD) according to the DSM-5 (American Psychiatric Association, 2013)^[2]^ and pervasive developmental disorder-not otherwise specified (PDD-NOS) according to the DSM-4 (American Psychiatric Association, 1994), excluding studies consisting solely of Rett syndrome diagnoses^[3]^. The inclusion of these various ASD diagnostic criteria was aimed at ensuring a comprehensive search of the DSM-5 standards. Studies were included if the mean age of the participants ranged from 3 to 12 years, even if some individuals were outside this age range.

(2)Intervention: Organized physical activity (OPA) is defined as formal and structured training sessions delivered by a coach (adult) through either paid or voluntary means in organizations, such as clubs, communities, schools, or educational institutions^[4][5][6]^. This review focuses on OPA and examines the latest research on group-based programs, such as Group-Based Organized Physical Activity (GBOPA). This study stipulated that each mentor or volunteer involved in the intervention must engage two or more participants to conduct the activity in a group setting. Studies will not be accepted if issues related to intervention allocation (such as group allocation) are not addressed in advance or if the intervention is conducted on a one-on-one basis. A minimum intervention duration of 30 min per session was established as an inclusion criterion on the basis of the structured format of group activities for children with special needs, which occurred at least once a week^[7]^. This timeframe aligns with the recommended guidelines for structured adult-led PA. Previous research has demonstrated that a school-based group physical activity program of 30 min per week is feasible for children with intellectual disabilities^[8]^.

(3)Control group: Organized physical activity (OPA) refers to structured physical activity programs in educational institutions, sports clubs, or other adult-led settings^[48]^. Numerous studies have demonstrated that such structured interventions help children develop positive social adaptation skills^[15][49][50]^. Since GBOPA is an extended development of OPA, with commonalities in the form of intervention organization, in order to prevent the overlapping effects between different interventions from interfering with the effect size of social abilities (the outcome variable of this study), control groups in the included studies must not be exposed to OPA programs. Additionally, studies utilizing control groups engaged in alternative group physical activities or social skills interventions were excluded; however, studies involving individual exercise or daily activities (e.g., psychotherapy or educational programs) were permitted.

(4)Outcome: The outcome indicators in this study was social abilities. Any assessments of self-reported or clinical ratings of social abilities in children and adolescents with autism spectrum disorder (ASD) were included. However, "empathy" was excluded from the assessment of social abilities because it encompasses areas beyond social abilities, such as emotional empathy and cognitive empathy^[9]^. Consequently, we excluded the empathy subscale when incorporating the Social Skills Improvement System (SSIS) questionnaire to ensure the authenticity and reliability of the results.

(5)Study design: This review included only experimental studies with pre- and post-test assessments, and only RCTs and quasi-experimental studies were included.

(6)Studies were written in English.

(7)Studies were published in peer-reviewed journals.

(8)Studies reported sufficient information to compute effect size.

(9)Studies involved human participants.

**1.3 Data Extraction**

A literature search was conducted in two stages. In the first stage, two researchers (JH and YG) identified the search keywords and used Rayyan to screen the literature according to the eligibility criteria. Upon completion of the screening, any disagreements (less than 1%) were discussed, and a consensus was reached. In the second stage, a third researcher (LZ) reviewed the full texts of the identified articles and, in collaboration with the second researcher (YG), determined whether any articles could be excluded and reached a consensus. Data extraction was performed independently by two researchers (JH and YG), who achieved a consistency of 94.85%. For any inconsistencies, a consensus was reached after discussion with a third researcher (LZ). We extracted the study identification data (i.e., author and year of publication) and moderating variables, which were categorized into population, intervention, control, and outcome variables on the basis of the PICO framework of evidence-based medicine.

**1.3.1 Population**

**Age** (categorical variable) refers to the mean age of the training participants. Children in different age groups have varying motivations and physical activity levels^[43][44]^. Therefore, individuals of different ages may experience different benefits from GBOPA^[45]^. In this study, age was converted from a continuous variable to a categorical variable on the basis of Erikson's eight stages of psychosocial development and the explanations by Estrugo et al. regarding the motivational, cognitive, and social cognitive mechanisms of social skills in both autism and typical development^[10][11]^. This framework divides autistic children into three developmental stages: early childhood (3-6 years), early school age (6-8.5 years), and preadolescence (8.6-12 years).

**1.3.2 Interventiccon**

**The type of training task** (categorical variable) were classified into two categories: motor skill-based interventions^[12]^ and motor technology-based interventions^[13]^. These interventions target different abilities in autistic children, which may influence social abilities and lead to varying outcomes. Motor skill-based interventions aim to improve physical function and basic motor skills through structured sessions that vary in intensity, duration, and training methods, such as aerobic running and strength training^[14]^. In contrast, motor technology-based interventions utilize structured and varied training methods for specific motor skills (e.g., dance and soccer).

**Training frequency** (a categorical variable) was defined as the number of interventions administered per week. It was categorized because all the studies included in this review reported the number of sessions per week, with frequencies ranging from 1 to 5 sessions. Thus, training frequency was divided into four distinct categories: 1, 2, 3, 4 or 5 sessions per week.

**The time point of the test** (categorical variable) was categorized into two levels: posttest and follow-up. The effects of training often diminish over time; therefore, the timing of post-intervention testing may serve as a moderating variable.We classified tests conducted within 7 days as post-tests and those administered more than 30 days after the last training as follow-up. Additionally, some studies reported only that a post-test was conducted without specifying the exact timing; we classified these as post-tests, based on the original text^[16][17][18][19]^. One study defined the post-test as occurring to 6-8 weeks after the intervention, and we categorized it into the follow-up group^[20]^. Studies with an interval of more than 10 days but less than 30 days between post-test and intervention completion were marked as "NA, not applicable"^[21]^.

**Group size** (dichotomous variable) refers to the number of individuals in a training group during the training process. Research has shown that group-based social skills interventions (GSSIs) lead to moderate improvements in the social abilities of autistic children^[46]^. Therefore, we must consider that different teacher-to-child ratios during the intervention may result in varying benefits for autistic children.However, this measure is only an approximation for two reasons. First, some studies did not specify the actual group size but reported only the student-to-instructor ratio (e.g., the student-instructor ratio = 1:3); thus, we calculated the number of students on the basis of this ratio, with the assumption of one instructor^[22][23]^. Second, in some studies, the group size was increased incrementally (e.g., from 1:1 to 1:3), prompting us to record the final number of participants in multiple group activities^[24]^. Additionally, we analyzed the included literature and marked studies that reported interventions in group settings (without specifying the exact ratios) as "NA, not applicable." We employed a binary approach to rank the 49 effect sizes related to social abilities (excluding NA) and found that the median group size was 3 individuals. Consequently, studies with group sizes less than or equal to three were classified as small (k = 24), whereas those with group sizes more than three were classified as large (k = 27).

**1.3.3 Control**

**The control group** (categorical variable) was divided into active and passive control groups. In the active control group, participants received non-OPA training programs (such as art programs or psychotherapy) that aligned with the experimental group's countermeasures, in addition to their daily treatment (e.g., compulsory education or outpatient care)^[24][22][25]^. In contrast, the passive control group received no interventions beyond their daily treatment and only participated in the pre- and postassessments.

**1.3.4 Outcome**

**The type of social ability** (categorical variable) in the DSM-IV identifies the core characteristics of autism as encompassing three domains: social interaction, communication, and repetitive/restricted behaviors^[3]^. Numerous studies on autistic children have reported effect sizes related to communication; however, the results have shown inconsistencies^[5][26][27]^. Consequently, this study will utilize communication as one of the classification variables, on the basis of the DSM-IV and prior research, while categorizing the remaining areas under social functions. This approach aimed to provide an evidence-based reference for clinical practice.

### 1.4 Statistical Analysis

Effect sizes and variances are calculated in Excel, consistently with Aksayli et al^[28]^. Subsequent statistical analyses were conducted using the metafor package in R Version 4.4.0 (R Core Team 2024)^[29]^.

### 1.4.1 Effect size Calculation

The effect size used was Hedges’*g*^[30]^. The formula was$g=\frac{\left( M_{e\_post}-M_{e\_pre} \right)-(M_{c\_post}-M_{c\_pre})}{\mathrm{SD}_{pooled\_pre}}$× (1- $\frac{3}{4\times N-9}$), where Me_post and Me_pre are the mean performance of the experimental group at the post-test and pre-test, respectively; Mc_post and Mc_pre are the mean performance of the control group at the post-test and pre-test, respectively; SDpooled_pre is the pooled pre-test SDs in the experimental group and the control group; and N is the total sample size. The formula is the most appropriate way to calculate the standardized mean difference in intervention studies with a repeated-measure design^[31]^.

The most accurate formula for sampling error variance in repeated measures designs requires pre-post-test correlations^[32]^. However, the pre-post-test correlations are rarely provided in the included primary studies. Therefore, consistent with Aksayli et al. (2019), we used $\mathrm{Var}_{g}=(\frac{N}{N_{e}\times N_{c}}+\frac{d^{2}}{2\times N})\times{(1- \frac{3}{4\times N-9})}^{2}$ as an acceptable approximation.

### 1.4.2 Meta-analysis

In our data, some studies reported multiple outcome variables or simultaneously reported results for the active and passive control groups, leading to dependence between effect sizes. To retain all effect sizes to enhance statistical power and address the dependency of effect sizes, it is necessary to use a multi-level or multi-variate approach^[33][34][35]^. Since most studies did not report correlations between variables needed by multivariate meta-analysis, we opted for a multi-level meta-analysis rather than a multivariate one. This method accounts for three levels of variance among effect sizes: sampling variance (level 1), the variance between effect sizes from the same study (level 2), and variance between studies (level 3).

First, we used a three-level model to compute the overall effect size. Following recommendations from previous research^[33][36]^, we employed the "t" test and “REML” (Restricted Maximum Likelihood) method when using the rma.mv function. Because the included studies were inconsistent with participant characteristics and intervention features, and we aimed to generalize the results to a broader population, we employed a random effects model, according to the suggestion of model selection^[30]^.

Secondly, we conducted an influence analysis to identify influential cases and assess their potential impact on the results^[29]^. An influential case was defined as the effect size whose exclusion from the analysis leads to considerable changes in the fitted model^[29]^. The meta-analytic models were thus run both with and without influential effect sizes.

Thirdly, we employed the Q statistics to examine the overall heterogeneity. Cochran’s Q statistic is calculated as the sum of the squared deviations of each effect size from the overall effect size, weighted by the inverse of the variance^[42]^. Also, we used I2 to estimate the heterogeneity within levels 1, 2, and 3, assessing the distribution of variance across levels^[36]^. In addition, we employed two log-likelihood ratio tests to assess whether the three-level model represents the variability in our data better than a two-level model^[36]^. Specifically, we set the variance at level 2 to zero, then fitted a two-level model including only levels 3 and 1. Then, we compared the results of the full model (three-level model) and the two-level model (with level 2 variance set to zero). Afterward, we set the variance at level 3 to zero and again compared the results between the full model (three-level model) and the two-level model (with level 3 variance set to zero).

Fourthly, for moderator analyses, we used three-level mixed-effects models^[30]^. We used the Q test, an omnibus test, to determine if the subgroup differences are large enough not to be explainable by sampling error alone^[33]^.

Fifthly, a cubic spline model was selected for multivariate regression analysis to examine the nonlinear relationship between the variables and effect size, with identifiers used as random factors to account for dependencies. The training session (continuous variable) refers to the total number of training sessions conducted. Training duration (continuous variable) was defined as the number of training sessions multiplied by the duration of each session, representing the total time participants were required to complete the training. We employed cubic spline linear regression models with three, four, and five knots, and compared these models using likelihood ratio tests to identify the best fit^[47]^.

Lastly, to assess publication bias, we employed the funnel plot^[37]^, Egger’s regression test (Egger et al., 1997), and trim-and-fill^[38]^. The funnel plot and trim-and-fill are based on a two-level model where all effect sizes are treated as "independent," as the two methods have not yet been extended to multi-level models^[39][40]^. Egger’s regression test simultaneously reported both the two and three-level models. In the three-level model, we utilized the predictor recommended by previous studies, which is 2√N, where N is the total sample size of the study^[39][40]^.

To comprehensively assess the intervention effects of GBOPA, we considered multiple outcome variables and types of control groups. For social abilities, we first disregarded differences in the type of outcome variable and differences in the control group and calculated the overall effect size by aggregating all effect sizes to assess whether GBOPA transfers to social ablilties generically. Subsequently, to systematically clarify which outcome variables were influenced by GBOPA, referring to a previous meta-analysis^[41]^, we examined the overall effect of different social abilities outcomes in the passive and active control groups. This was done because only the active control group could provide convincing evidence of specific benefits from GBOPA.

**2 Supplementary Result**

### 2.1 Characteristics of the studies

**Table S2** Characteristics of studies and effect sizes included in the meta-analysis of social abilities

| **Study** | **Study**  **design** | **N** | **Age** | **Group**  **size** | **Control group** | **Task** | **Sessions** | **Duration** | **Per week** | **Test_Time** | **Outcome** | **Assessment** | **g** |
| --- | --- | --- | --- | --- | --- | --- | --- | --- | --- | --- | --- | --- | --- |
| (Ebony et al., 2024)-1 | RCT | 27 | 9 | NR | active | technology | 10 | 600 | one | NR | communication | SRS | -0.0096 |
| (Ebony et al., 2024)-2 | RCT | 27 | 9 | NR | active | technology | 10 | 600 | one | NR | social function | SRS | -0.0249 |
| (Toscano et al., 2022)-1 | CT | 167 | 7.9 | NR | active | skill | 96 | 3840 | two | posttest | social function | ATA: Social interaction | 0.4625 |
| (Toscano et al., 2022)-2 | CT | 189 | 7.9 | NR | active | skill | 96 | 3840 | two | posttest | social function | ATA: Social interaction | 0.2741 |
| (Toscano et al., 2022)-3 | CT | 189 | 7.9 | NR | active | skill | 96 | 3840 | two | posttest | communication | ATA: verbal stereotype | 0.1132 |
| (Toscano et al., 2022)-4 | CT | 167 | 7.9 | NR | active | skill | 96 | 3840 | two | posttest | communication | ATA: verbal stereotype | 0.0742 |
| (Lavinia et al., 2022)-1 | RCT | 27 | 9.96 | 8 | passive | technology | 24 | 960 | one | posttest | social function | FIM: Social interaction | 2.9208 |
| (Lavinia et al., 2022)-2 | RCT | 27 | 9.96 | 8 | passive | technology | 24 | 960 | one | posttest | communication | ABC: Language behavior | 2.9086 |
| (Lavinia et al., 2022)-3 | RCT | 27 | 9.96 | 8 | passive | technology | 24 | 960 | one | posttest | communication | CARS: Verbal communication | 2.3057 |
| (Lavinia et al., 2022)-4 | RCT | 27 | 9.96 | 8 | passive | technology | 24 | 960 | one | posttest | communication | CARS: Non-Verbal communication | 1.3648 |
| (Phung et al., 2021)-1 | RCT | 34 | 9.1 | 7 | passive | technology | 26 | 1170 | two | posttest | social function | SSIS：Problem Behaviors Scale | 0.963 |
| (Phung et al., 2021)-2 | RCT | 34 | 9.1 | 7 | passive | technology | 26 | 1170 | two | posttest | social function | SSIS：Social Skills Scale | 0.558 |
| (Howells et al., 2020)-1 | CT | 40 | 7.98 | NR | passive | technology | 14 | 1200 | one | follow-up | social function | CBCL： Social problems | 0.6581 |
| (Howells et al., 2020)-2 | CT | 40 | 7.98 | NR | passive | technology | 14 | 1200 | one | follow-up | communication | VABS-3：Communication | 0.1006 |
| (Howells et al., 2020)-3 | CT | 40 | 7.98 | NR | passive | technology | 14 | 1200 | one | follow-up | social function | VABS-3：Socialization | -0.0186 |
| (Zhao et al., 2018)-1 | QE | 41 | 6.14 | 5 | active | skill | 24 | 1440 | two | posttest | social function | ABLLS-R：social interaction | 2.2071 |
| (Zhao et al., 2018)-2 | QE | 41 | 6.14 | 5 | active | skill | 24 | 1440 | two | posttest | communication | SSIS：Communication | 0.9079 |
| (Zhao et al., 2018)-3 | QE | 41 | 6.14 | 5 | active | skill | 12 | 720 | two | posttest | social function | ABLLS-R：social interaction | 0.901 |
| (Zhao et al., 2018)-4 | QE | 41 | 6.14 | 5 | active | skill | 24 | 1440 | two | posttest | social function | SSIS:Responsibility | 0.7339 |
| (Zhao et al., 2018)-5 | QE | 41 | 6.14 | 5 | active | skill | 24 | 1440 | two | posttest | social function | SSIS:Engagement | 0.597 |
| (Zhao et al., 2018)-6 | QE | 41 | 6.14 | 5 | active | skill | 12 | 720 | two | posttest | social function | SSIS:Engagement | 0.567 |
| (Zhao et al., 2018)-7 | QE | 41 | 6.14 | 5 | active | skill | 12 | 720 | two | posttest | communication | SSIS：Communication | 0.5428 |
| (Zhao et al., 2018)-8 | QE | 41 | 6.14 | 5 | active | skill | 24 | 1440 | two | posttest | social function | SSIS:Cooperation | 0.5348 |
| **Study** | **Study**  **design** | **N** | **Age** | **Group**  **size** | **Control group** | **Task** | **Sessions** | **Duration** | **Per week** | **Test_Time** | **Outcome** | **Assessment** | **g** |
| (Zhao et al., 2018)-9 | QE | 41 | 6.14 | 5 | active | skill | 24 | 1440 | two | posttest | social function | SSIS:Self-control | 0.5328 |
| (Zhao et al., 2018)-10 | QE | 41 | 6.14 | 5 | active | skill | 12 | 720 | two | posttest | social function | SSIS:Cooperation | 0.449 |
| (Zhao et al., 2018)-11 | QE | 41 | 6.14 | 5 | active | skill | 12 | 720 | two | posttest | social function | SSIS:Responsibility | 0.3384 |
| (Zhao et al., 2018)-12 | QE | 41 | 6.14 | 5 | active | skill | 12 | 720 | two | posttest | social function | SSIS:Self-control | 0.2304 |
| (Zhao et al., 2018)-13 | QE | 41 | 6.14 | 5 | active | skill | 12 | 720 | two | posttest | social function | SSIS:Assertion | 0.0861 |
| (Zhao et al., 2018)-14 | QE | 41 | 6.14 | 5 | active | skill | 24 | 1440 | two | posttest | social function | SSIS:Assertion | -0.2636 |
| (Borgi et al., 2015)-1 | RCT | 26 | 9.20 | 3 | passive | technology | 25 | 1625 | one | posttest | communication | VABS：Communication | 0.3089 |
| (Borgi et al., 2015)-2 | RCT | 26 | 9.20 | 3 | passive | technology | 25 | 1625 | one | posttest | social function | VABS：socialization | 0.2734 |
| (Movahedi et al., 2013)-1 | RCT | 26 | 9.54 | 13 | active | technology | 56 | 4080 | four | posttest | social function | GARS-2:social interaction | 1.122 |
| (Movahedi et al., 2013)-2 | RCT | 26 | 9.54 | 13 | active | technology | 56 | 4080 | four | follow-up | social function | GARS-2:social interaction | 0.7265 |
| (Gabriels et al., 2012)-1 | CT | 56 | 8.7 | 4 | passive | technology | 10 | 600 | one | posttest | social function | VABS-II：social | 0.4717 |
| (Gabriels et al., 2012)-2 | CT | 56 | 8.7 | 4 | passive | technology | 10 | 600 | one | posttest | communication | VABS-II：communication | 0.343 |
| (Bass et al., 2009)-1 | CT | 22 | 6.95 | NR | passive | technology | 12 | 720 | one | posttest | social function | SRS | 0.5097 |
| (Zachor et al., 2017)-1 | CT | 51 | 5.6 | 2 | passive | skill | 13 | 390 | one | posttest | social function | SRS:Autistic mannerisms | 0.6789 |
| (Zachor et al., 2017)-2 | CT | 51 | 5.6 | 2 | passive | skill | 13 | 390 | one | posttest | social function | SRS:Social cognition | 0.5586 |
| (Zachor et al., 2017)-3 | CT | 51 | 5.6 | 2 | passive | skill | 13 | 390 | one | posttest | communication | SRS:Social communication | 0.3695 |
| (Zachor et al., 2017)-4 | CT | 51 | 5.6 | 2 | passive | skill | 13 | 390 | one | posttest | social function | SRS: Social awareness | 0.3107 |
| (Zachor et al., 2017)-5 | CT | 51 | 5.6 | 2 | passive | skill | 13 | 390 | one | posttest | social function | SRS: Social motivation | 0.2814 |
| (Gabriels et al., 2015)-1 | RCT | 97 | 10.5 | 3 | active | technology | 10 | 450 | one | posttest | communication | SRS：Social Communication | 0.6316 |
| (Gabriels et al., 2015)-2 | RCT | 97 | 10.5 | 3 | active | technology | 10 | 450 | one | posttest | social function | SRS：Social Cognition | 0.4429 |
| (Gabriels et al., 2015)-3 | RCT | 97 | 10.5 | 3 | active | technology | 10 | 450 | one | posttest | social function | SRS：Social Motivation | 0.3335 |
| (Gabriels et al., 2015)-4 | RCT | 97 | 10.5 | 3 | active | technology | 10 | 450 | one | posttest | communication | SALT：Number words used | 0.2644 |
| (Gabriels et al., 2015)-5 | RCT | 97 | 10.5 | 3 | active | technology | 10 | 450 | one | posttest | social function | SRS：Social Awareness | 0.2105 |
| (Gabriels et al., 2015)-6 | RCT | 97 | 10.5 | 3 | active | technology | 10 | 450 | one | posttest | communication | SALT：Number different words used | 0.2012 |
| (Gabriels et al., 2015)-7 | RCT | 97 | 10.5 | 3 | active | technology | 10 | 450 | one | posttest | social function | VABS：Socialization raw score | 0.1691 |
| (Gabriels et al., 2015)-8 | RCT | 97 | 10.5 | 3 | active | technology | 10 | 450 | one | posttest | social function | SRS：Autistic Mannerisms | 0.1583 |
| (Gabriels et al., 2015)-9 | RCT | 97 | 10.5 | 3 | active | technology | 10 | 450 | one | posttest | communication | VABS：Communication raw score | -0.023 |
| (Bahrami et al., 2016)-1 | RCT | 22 | 9.2 | NR | passive | skill | 56 | 4080 | four | posttest | communication | GARS-2:communication subscale | 0.6557 |
| **Study** | **Study**  **design** | **N** | **Age** | **Group**  **size** | **Control group** | **Task** | **Sessions** | **Duration** | **Per week** | **Test_Time** | **Outcome** | **Assessment** | **g** |
| (Bahrami et al., 2016)-2 | RCT | 22 | 9.2 | NR | passive | skill | 56 | 4080 | four | follow-up | communication | GARS-2: communication subscale | 0.5247 |
| (Caputo et al., 2018)-1 | CT | 26 | 8.3 | 3 | active | technology | 96 | 4320 | two | posttest | communication | VABS:Communication | 0.4706 |
| (Caputo et al., 2018)-2 | CT | 26 | 8.3 | 3 | active | technology | 96 | 4320 | two | posttest | social function | VABS: Social abilities | 0.3609 |
| (Caputo et al., 2018)-3 | CT | 26 | 8.3 | 3 | active | technology | 96 | 4320 | two | posttest | communication | CARS: Verbal communication | 0.2721 |
| (Caputo et al., 2018)-4 | CT | 26 | 8.3 | 3 | active | technology | 96 | 4320 | two | posttest | communication | CARS: Non-verbal communication | 0.0915 |
| (Sotoodeh et al., 2017)-1 | RCT | 29 | 10.8 | 5 | passive | technology | 24 | 720 | three | posttest | social function | ATEC: Sociability | 0.2909 |
| (Sotoodeh et al., 2017)-2 | RCT | 29 | 10.8 | 5 | passive | technology | 24 | 720 | three | posttest | communication | ATEC: Speech and Language | 0.199 |
| (Zhao et al., 2021)-1 | QE | 61 | 7.06 | NR | passive | technology | 32 | 1920 | two | posttest | social function | ABLLS-R | 2.2453 |
| (Zhao et al., 2021)-2 | QE | 61 | 7.06 | NR | passive | technology | 32 | 1920 | two | posttest | communication | SSIS-RS:Communication | 0.8959 |
| (Zhao et al., 2021)-3 | QE | 61 | 7.06 | NR | passive | technology | 32 | 1920 | two | posttest | communication | Says thank you | 0.7039 |
| (Zhao et al., 2021)-4 | QE | 61 | 7.06 | NR | passive | technology | 16 | 960 | two | posttest | social function | ABLLS-R | 0.6653 |
| (Zhao et al., 2021)-5 | QE | 61 | 7.06 | NR | passive | technology | 32 | 1920 | two | posttest | social function | SSIS-RS:Self-control | 0.6244 |
| (Zhao et al., 2021)-6 | QE | 61 | 7.06 | NR | passive | technology | 32 | 1920 | two | posttest | communication | Says please | 0.5571 |
| (Zhao et al., 2021)-7 | QE | 61 | 7.06 | NR | passive | technology | 16 | 960 | two | posttest | communication | SSIS-RS:Communication | 0.5474 |
| (Zhao et al., 2021)-8 | QE | 61 | 7.06 | NR | passive | technology | 32 | 1920 | two | posttest | communication | Uses gestures or body appropriately with others | 0.4988 |
| (Zhao et al., 2021)-9 | QE | 61 | 7.06 | NR | passive | technology | 32 | 1920 | two | posttest | social function | SSIS-RS:Responsibility | 0.4303 |
| (Zhao et al., 2021)-10 | QE | 61 | 7.06 | NR | passive | technology | 32 | 1920 | two | posttest | communication | Makes eye contact when talking | 0.4254 |
| (Zhao et al., 2021)-11 | QE | 61 | 7.06 | NR | passive | technology | 16 | 960 | two | posttest | communication | Says thank you | 0.4166 |
| (Zhao et al., 2021)-12 | QE | 61 | 7.06 | NR | passive | technology | 16 | 960 | two | posttest | communication | Uses gestures or body appropriately with others | 0.4048 |
| (Zhao et al., 2021)-13 | QE | 61 | 7.06 | NR | passive | technology | 32 | 1920 | two | posttest | social function | SSIS-RS:Engagement | 0.3476 |
| (Zhao et al., 2021)-14 | QE | 61 | 7.06 | NR | passive | technology | 16 | 960 | two | posttest | social function | SSIS-RS:Engagement | 0.3366 |
| (Zhao et al., 2021)-15 | QE | 61 | 7.06 | NR | passive | technology | 32 | 1920 | two | posttest | social function | SSIS-RS:Cooperation | 0.3138 |
| (Zhao et al., 2021)-16 | QE | 61 | 7.06 | NR | passive | technology | 16 | 960 | two | posttest | social function | SSIS-RS:Self-control | 0.2761 |
| (Zhao et al., 2021)-17 | QE | 61 | 7.06 | NR | passive | technology | 32 | 1920 | two | posttest | social function | SSIS-RS:Self-control | 0.2594 |
| (Zhao et al., 2021)-18 | QE | 61 | 7.06 | NR | passive | technology | 16 | 960 | two | posttest | communication | Says please | 0.2559 |
| (Zhao et al., 2021)-19 | QE | 61 | 7.06 | NR | passive | technology | 32 | 1920 | two | posttest | communication | Take turns in conversations | 0.2468 |
| **Study** | **Study**  **design** | **N** | **Age** | **Group**  **size** | **Control group** | **Task** | **Sessions** | **Duration** | **Per week** | **Test_Time** | **Outcome** | **Assessment** | **g** |
| (Zhao et al., 2021)-20 | QE | 61 | 7.06 | NR | passive | technology | 16 | 960 | two | posttest | social function | SSIS-RS:Cooperation | 0.2295 |
| (Zhao et al., 2021)-21 | QE | 61 | 7.06 | NR | passive | technology | 16 | 960 | two | posttest | communication | Take turns in conversations | 0.2252 |
| (Zhao et al., 2021)-22 | QE | 61 | 7.06 | NR | passive | technology | 32 | 1920 | two | posttest | communication | Responds well when others start a conversation | 0.1814 |
| (Zhao et al., 2021)-23 | QE | 61 | 7.06 | NR | passive | technology | 16 | 960 | two | posttest | communication | Speaks in appropriate tone of voice | 0.1636 |
| (Zhao et al., 2021)-24 | QE | 61 | 7.06 | NR | passive | technology | 16 | 960 | two | posttest | communication | Makes eye contact when talking | 0.145 |
| (Zhao et al., 2021)-25 | QE | 61 | 7.06 | NR | passive | technology | 16 | 960 | two | posttest | social function | SSIS-RS:Assertion | 0.1155 |
| (Zhao et al., 2021)-26 | QE | 61 | 7.06 | NR | passive | technology | 16 | 960 | two | posttest | communication | Responds well when others start a conversation | 0.0645 |
| (Zhao et al., 2021)-27 | QE | 61 | 7.06 | NR | passive | technology | 16 | 960 | two | posttest | social function | SSIS-RS:Responsibility | 0.0421 |
| (Zhao et al., 2021)-28 | QE | 61 | 7.06 | NR | passive | technology | 32 | 1920 | two | posttest | communication | Speaks in appropriate tone of voice | 0.0368 |
| (Najafabadi et al., 2018)-1 | RCT | 26 | 7.08 | 3 | passive | technology | 36 | 1440 | three | posttest | social function | GARS-2: Social interaction | 0.9815 |
| (Najafabadi et al., 2018)-2 | RCT | 26 | 7.08 | 3 | passive | skill | 36 | 1440 | three | posttest | social function | ATEC: Sociability | 0.8827 |
| (García-Gómez et al.,2013)-1 | CT | 16 | 9.2 | 4 | NR | technology | 5 | 150 | two | posttest | social function | BASC-T:Social skills | 0.4637 |
| (Özcan et al., 2024)-1 | RCT | 34 | 4.83 | NR | passive | skill | 24 | 1440 | two | posttest | social function | SSRS-Collaboration | 0.8965 |
| (Özcan et al., 2024)-2 | RCT | 34 | 4.83 | NR | passive | skill | 24 | 1440 | two | posttest | social function | SSRS-Internalizing | 0.8795 |
| (Özcan et al., 2024)-3 | RCT | 34 | 4.83 | NR | passive | skill | 24 | 1440 | two | posttest | social function | SSRS-Self-control | 0.7766 |
| (Özcan et al., 2024)-4 | RCT | 34 | 4.83 | NR | passive | skill | 24 | 1440 | two | posttest | social function | SSRS-Externalizing | 0.772 |
| (Özcan et al., 2024)-5 | RCT | 34 | 4.83 | NR | passive | skill | 24 | 1440 | two | posttest | social function | GARS-Social interaction | 0.5317 |
| (Özcan et al., 2024)-6 | RCT | 34 | 4.83 | NR | passive | skill | 24 | 1440 | two | posttest | social function | SSRS-Self-expression | 0.4723 |
| (Özcan et al., 2024)-7 | RCT | 34 | 4.83 | NR | passive | skill | 24 | 1440 | two | posttest | communication | GARS-Communication | 0.1428 |
| (Ju et al., 2024)-1 | RCT | 17 | 11.11 | NR | passive | technology | 24 | 1140 | three | follow-up | social function | ABC-Social Withdrawal | 0.9181 |
| (Ju et al., 2024)-2 | RCT | 17 | 11.11 | NR | passive | technology | 24 | 1140 | three | posttest | social function | ABC-Social Withdrawal | 0.8837 |
| (Ju et al., 2024)-3 | RCT | 17 | 11.11 | NR | passive | technology | 12 | 570 | three | posttest | social function | ABC-Social Withdrawal | 0.4947 |
| (Ju et al., 2024)-4 | RCT | 17 | 11.11 | NR | passive | technology | 24 | 1140 | three | posttest | communication | ABC-Inappropriate Speech | 0.1541 |
| (Ju et al., 2024)-5 | RCT | 17 | 11.11 | NR | passive | technology | 24 | 1140 | three | follow-up | communication | ABC-Inappropriate Speech | 0.0309 |
| (Ju et al., 2024)-6 | RCT | 17 | 11.11 | NR | passive | technology | 12 | 570 | three | posttest | communication | ABC-Inappropriate Speech | -0.1747 |
| (Cai et al., 2020a)-1 | QE | 29 | 4.68 | NR | active | technology | 60 | 2400 | five | posttest | social function | SRS:Social cognition | 1.144 |
| **Study** | **Study**  **design** | **N** | **Age** | **Group**  **size** | **Control group** | **Task** | **Sessions** | **Duration** | **Per week** | **Test_Time** | **Outcome** | **Assessment** | **g** |
| (Cai et al., 2020a)-2 | QE | 29 | 4.68 | NR | active | technology | 60 | 2400 | five | posttest | social function | SRS:Autistic mannerisms | 0.8971 |
| (Cai et al., 2020a)-3 | QE | 29 | 4.68 | NR | active | technology | 60 | 2400 | five | posttest | communication | SRS:Social communication | 0.8608 |
| (Cai et al., 2020a)-4 | QE | 29 | 4.68 | NR | active | technology | 60 | 2400 | five | posttest | social function | SRS:Social motivation | 0.5136 |
| (Cai et al., 2020a)-5 | QE | 29 | 4.68 | NR | active | technology | 60 | 2400 | five | posttest | social function | SRS: Social awareness | 0.4461 |
| (Cai et al., 2020b)-1 | QE | 30 | 4.56 | NR | active | technology | 60 | 2400 | five | posttest | social function | SRS:Social cognition | 1.0779 |
| (Cai et al., 2020b)-2 | QE | 30 | 4.56 | NR | active | technology | 60 | 2400 | five | posttest | communication | SRS:Social communication | 1.0098 |
| (Cai et al., 2020b)-3 | QE | 30 | 4.56 | NR | active | technology | 60 | 2400 | five | posttest | social function | SRS:Autistic mannerisms | 1.0032 |
| (Cai et al., 2020b)-4 | QE | 30 | 4.56 | NR | active | technology | 60 | 2400 | five | posttest | social function | SRS: Social awareness | 0.6431 |
| (Cai et al., 2020b)-5 | QE | 30 | 4.56 | NR | active | technology | 60 | 2400 | five | posttest | social function | SRS:Social motivation | 0.5749 |
| (Zhou et al., 2024)-1 | QE | 58 | 6.4 | NR | active | technology | 60 | 2400 | five | posttest | communication | SRS:Social communication | 0.7119 |
| (Zhou et al., 2024)-2 | QE | 58 | 6.4 | NR | active | technology | 60 | 2400 | five | posttest | social function | SRS:Autistic mannerisms | 0.6406 |
| (Zhou et al., 2024)-3 | QE | 58 | 6.4 | NR | active | technology | 60 | 2400 | five | posttest | social function | SRS:Social motivation | 0.5291 |
| (Zhou et al., 2024)-4 | QE | 58 | 6.4 | NR | active | technology | 60 | 2400 | five | posttest | social function | SRS:Social cognition | 0.3352 |
| (Zhou et al., 2024)-5 | QE | 58 | 6.4 | NR | active | technology | 60 | 2400 | five | posttest | social function | SRS: Social awareness | 0.1914 |
| (Broupi et al., 2023)-1 | QE | 18 | NR | 3 | passive | technology | 36 | 2160 | three | posttest | social function | SCQ: Social behavior | 1.769 |
| (Broupi et al., 2023)-2 | QE | 18 | NR | 3 | passive | technology | 36 | 2160 | three | posttest | communication | SCQ: Communication | 1.136 |

Note: “NR” represents “Not Reported”; “Duration” represents “Training Duration,” and it is measured in minutes; the numbering following the references represents the order of the effect sizes within the study. For example, (Gabriels et al., 2015)-4 signifies the fourth effect size that the study contributed; SRS = The Social Responsiveness Scale; ATA = Autistic Traits Assessment Scale; FIM = Functional Independence Measure; ABC = Autism Behavior Checklist; CARS = The Childhood Autism Rating Scale; SSIS = Social Skills Improvement System; CBCL = Child Behaviour Checklist-caregiver version; VABS = Vineland Adaptive Behaviour Scale; ABLLS-R = The Assessment of Basic Language and Learning Skills; SSIS = Social Skills Improvement System; VABS = Vineland Adaptive Behaviour Scale; SALT = Systematic Analysis of Language Transcripts; ATEC = The Autism Treatment Evaluation Checklist; BASC = ;Behavior Assessment System for Children; SSIS-RS = Social Skills Improvement System Rating Scales; SSRS = Social Skills Rating System; GARS = The Gilliam Autistic Disorder Rating Scale; SCQ = The Social Communication Questionnaire.

**Table S3** Sensitivity analysis (Retaining outliers vs. Excluding outliers)

|  | **Retaining outliers** | | | |  | **Excluding outliers** | | | |
| --- | --- | --- | --- | --- | --- | --- | --- | --- | --- |
| **Outcome** | **Hedge’s [95%CI]** | ***p-value*** | ***Q*** | **PI** |  | **Hedge’s [95%CI]** | ***p-value*** | ***Q*** | **PI** |
| **Social abilities** | Level2: 0.49 [0.42, 0.57] | <0.001 | 235.67*** | [-0.06, 1.05] |  | Level2: 0.40 [0.35, 0.45] | <0.001 | 114.84 | [0.30, 0.50] |
|  | Level3: 0.58 [0.40, 0.75] | <0.001 | 235.67*** | [-0.22, 1.37] |  | Level3: 0.48 [0.38, 0.59] | <0.001 | 114.84 | [0.12, 0.84] |
| Social function | Level2: 0.54 [0.44, 0.63] | <0.001 | 113.03*** | [0.01, 1.07] |  | Level2: 0.44 [0.37, 0.51] | <0.001 | 62.97 | [0.37, 0.51] |
|  | Level3: 0.54 [0.44, 0.64] | <0.001 | 113.03*** | [0.01, 1.07] |  | Level3: 0.50 [0.39, 0.60] | <0.001 | 62.97 | [0.21, 0.79] |
| Communication | Level2: 0.43 [0.30, 0.55] | <0.001 | 98.20*** | [-0.17, 1.03] |  | Level2: 0.34 [0.25, 0.44] | <0.001 | 48.07 | [0.12, 0.56] |
|  | Level3: 0.49 [0.25, 0.72] | <0.001 | 98.20*** | [-0.41, 1.38] |  | Level3: 0.37 [0.24, 0.51] | <0.001 | 48.07 | [0.04, 0.70] |

Note: Level 2 represents the two-level random-effects model; Level 3 represents the three-level random-effects model; CI represents the confidence interval; PI represents the prediction interval; Q represents Cochran’s Q statistic; *** p < 0.001.

**Table S4** GRADE level of evidence for this study’s findings

| **No.Studies** | | **Total N** | **k** | **Quality assessment** | | | | | **Hedge’s g**  **(95%CI)** | **Evidence Quality*** |
| --- | --- | --- | --- | --- | --- | --- | --- | --- | --- | --- |
|  |  |  |  | **Study limitations** | **Consistency** | **Directness** | **Precision** | **Publication bias** |  |  |
| **Social abilities** | | 1042 | 104 | ↓ 1 level^1)^ | No down | No down | No down | ↓ 1 level^3)^ | 0.48[0.38,0.59**]** | **⨁⨁○○**,low |
| 24(11 RCT, 13 Cohort) | |  |  |  |  |  |  |  |  |  |
| Social function | | 993 | 70 | ↓ 1 level^1)^ | No down | No down | No down | ↓ 1 level^3)^ | 0.50[0.39,0.60] | **⨁⨁○○**,low |
| 22(9 RCT, 12 Cohort) | |  |  |  |  |  |  |  |  |  |
| Communication | | 926 | 44 | ↓ 1 level^1)^ | No down | No down | No down | ↓ 1 level^3)^ | 0.37[0.24,0.51] | **⨁⨁○○**,low |
| 19(7 RCT, 12 Cohort) | |  |  |  |  |  |  |  |  |  |
| **Optimal dose** | Training session | | | ↓ 1 level^1)^ | No down | No down | ↓ 1 level^2)^ | No down | Exploratory analysis | **⨁⨁○○**,low |
|  | Training duration | | | ↓ 1 level^1)^ | No down | No down | ↓ 1 level^2)^ | No down | Exploratory analysis | **⨁⨁○○**,low |
| *GRADE: Quality of Clinical Evidence and Recommendation Levels:  High: The research team has strong confidence in the estimated effect size;  Moderate: The research team has moderate confidence in the estimated effect size;  Low: The research team has limited confidence in the estimated effect size;  Very low: The research team has very limited confidence in the estimated effect size. | | | | | | | | | | |

Note: 1) In studies associated with more than 95% of the effect sizes, the allocation concealment strategy used during the process from registration to intervention assignment was unclear. Additionally, in 23.5% of these studies, the overall dropout rate for interventions exceeded 20%, and over 50% did not employ a double-blind strategy. 2) The exploratory analysis led to imprecision in the data. 3) Evidence of publication bias was observed.

**2.2 Effect of GBOPA on social ability**

**Figure S1**


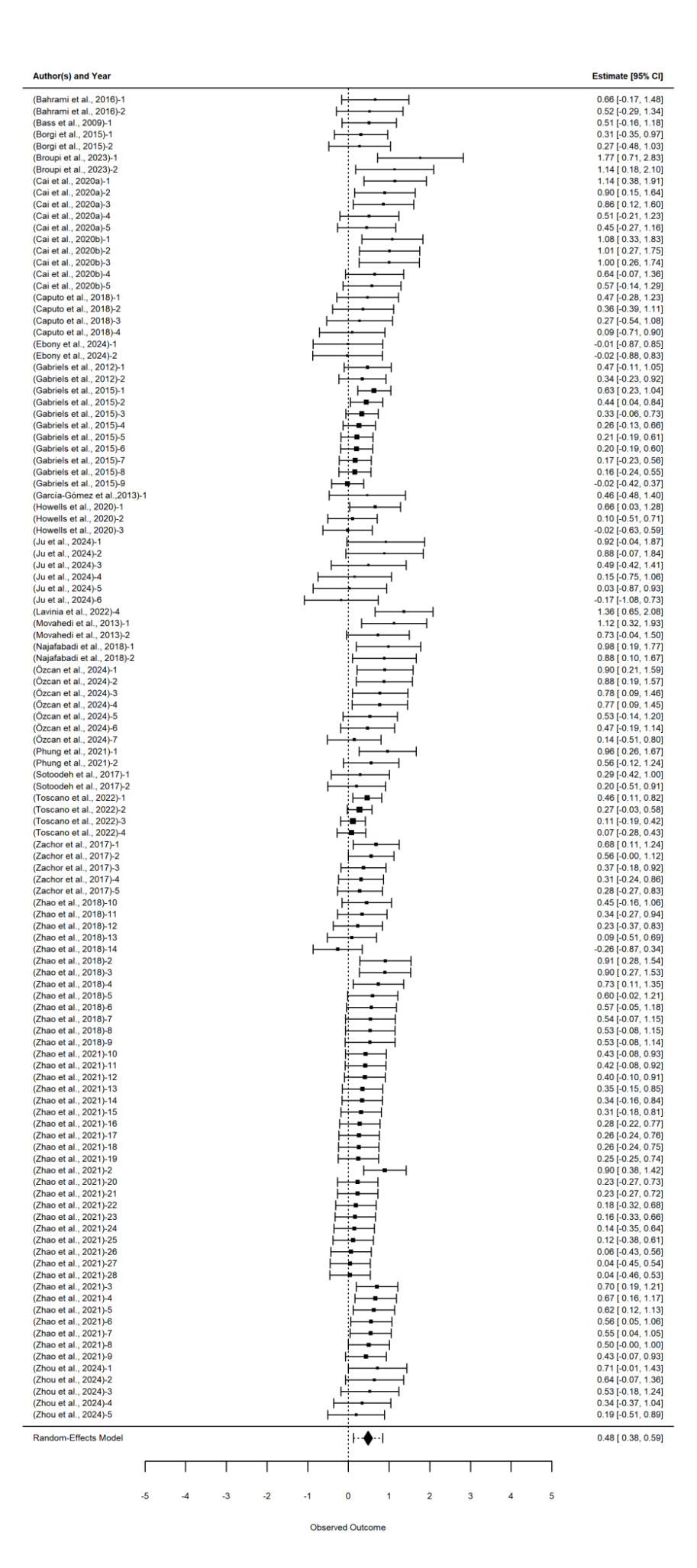


**Fig S1.** The forest plot of the effect of GBOPA on social ability (no outliers). Note: CI, confidence interval; RE model, random-effects model; the numbering following the references represents the order of the effect sizes within the study. For example, (Zachor et al., 2017)-4 signifies the fourth effect size that the study contributed.

### Figure S2


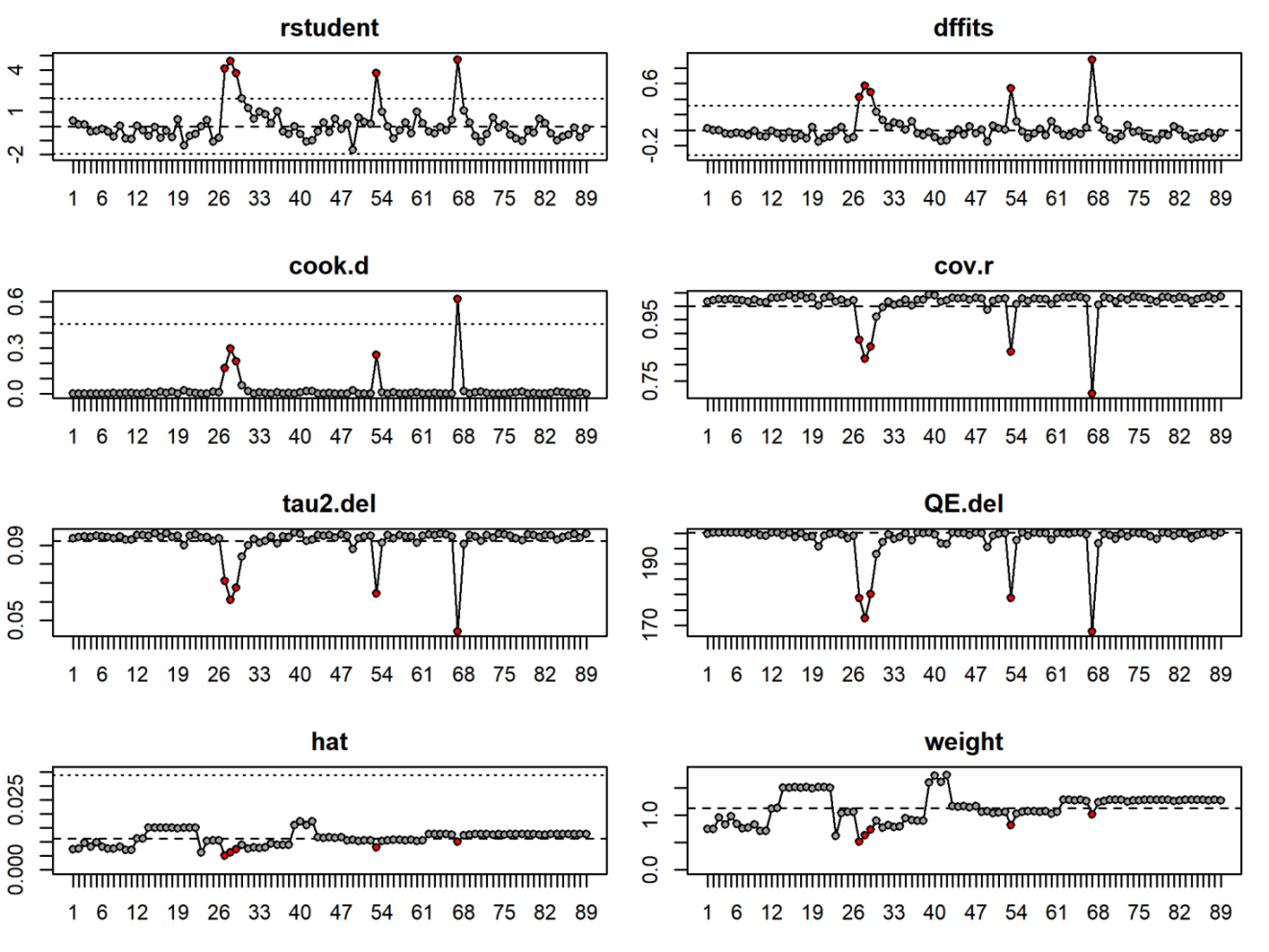


**Fig S2.** Results of the influence analysis, with influential cases in red.

### Figure S3

**
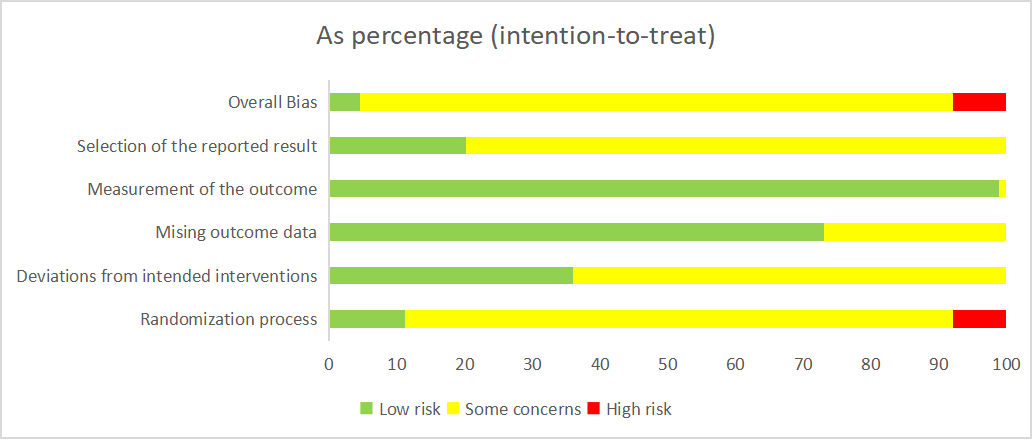
**

**Fig S3.** Risk of bias summary of the RoB2 assessments. It was created via robvis (McGuinness & Higgins, 2021)

### Figure S4


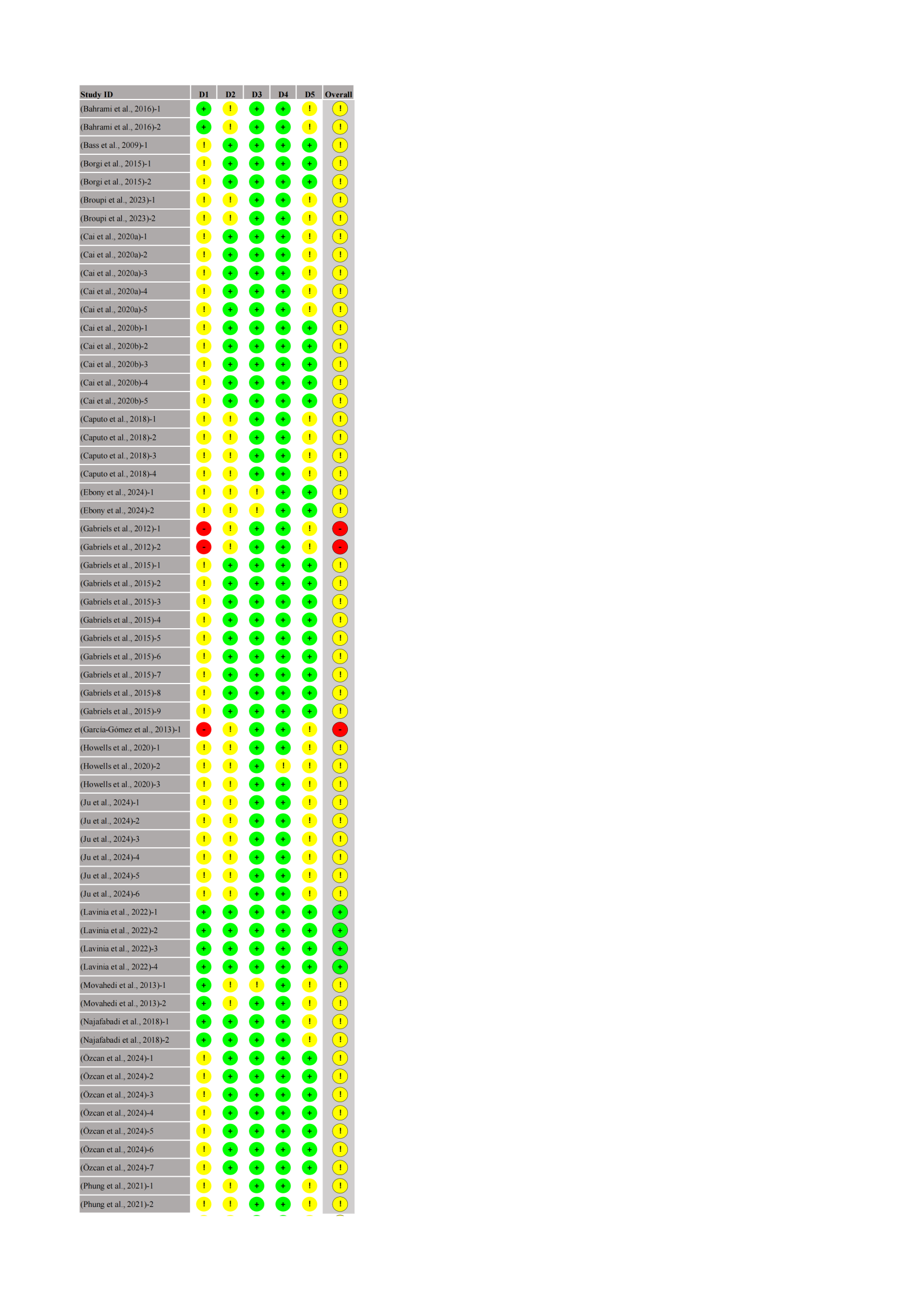

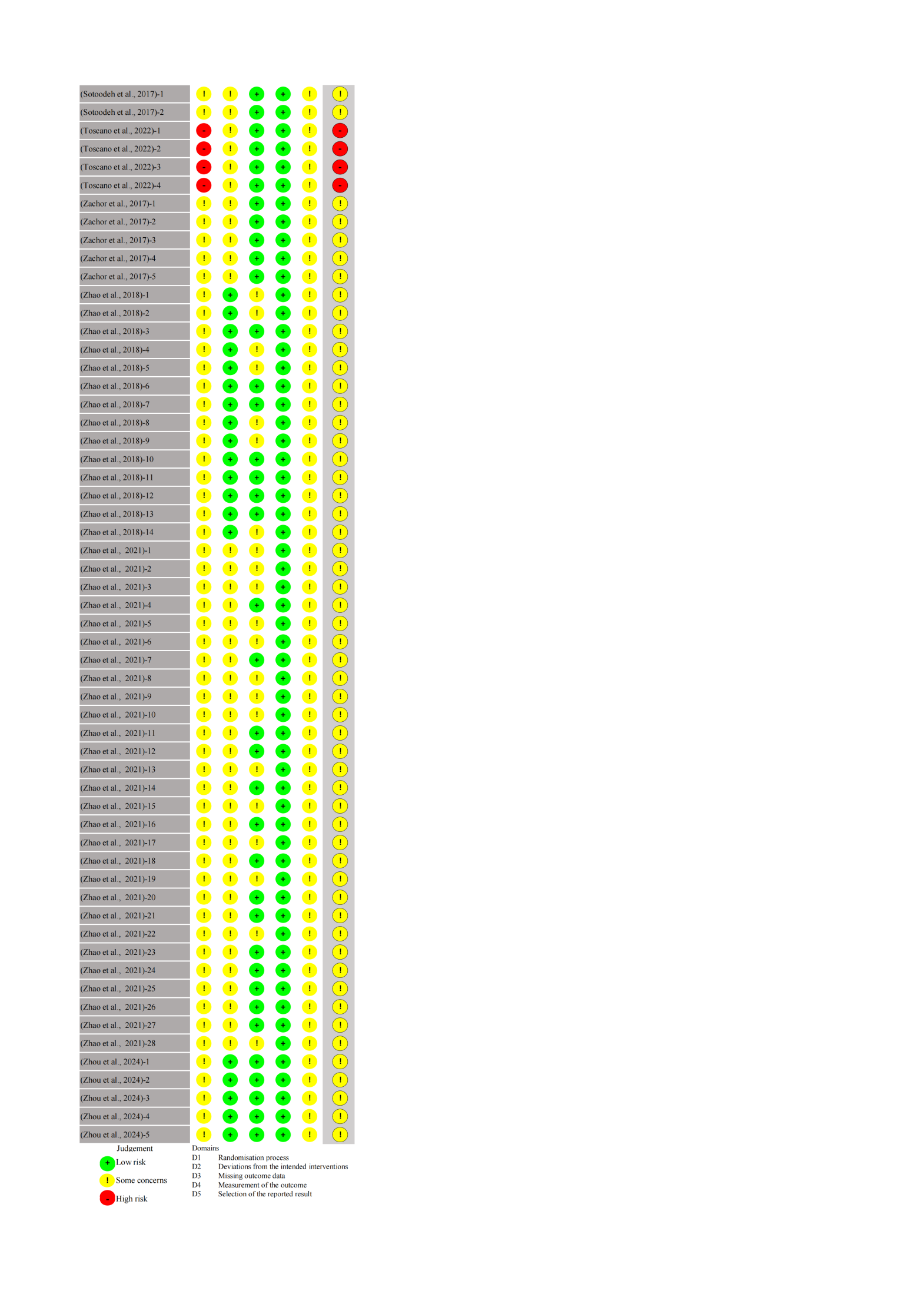


**Fig S4.** Risk of bias traffic light plot of RoB2 assessments. It was created via robvis (McGuinness & Higgins, 2021).

## **3 PRISMA 2020 Checklist**

| **Section and Topic** | **Item #** | **Checklist item** | **Location where item is reported** |
| --- | --- | --- | --- |
| **TITLE** | | |  |
| Title | 1 | Identify the report as a systematic review. | Lines 1-2  (manuscript) |
| **ABSTRACT** | | |  |
| Abstract | 2 | See the PRISMA 2020 for Abstracts checklist. | Lines 19-38  (manuscript) |
| **INTRODUCTION** | | |  |
| Rationale | 3 | Describe the rationale for the review in the context of existing knowledge. | Lines 44-93  (manuscript) |
| Objectives | 4 | Provide an explicit statement of the objective(s) or question(s) the review addresses. | Lines 44-93  (manuscript) |
| **METHODS** | | |  |
| Eligibility criteria | 5 | Specify the inclusion and exclusion criteria for the review and how studies were grouped for the syntheses. | Lines 9-47  (supplementary material) |
| Information sources | 6 | Specify all databases, registers, websites, organisations, reference lists and other sources searched or consulted to identify studies. Specify the date when each source was last searched or consulted. | Lines 95-98  (manuscript) |
| Search strategy | 7 | Present the full search strategies for all databases, registers and websites, including any filters and limits used. | Lines 5-8  (supplementary material) |
| Selection process | 8 | Specify the methods used to decide whether a study met the inclusion criteria of the review, including how many reviewers screened each record and each report retrieved, whether they worked independently, and if applicable, details of automation tools used in the process. | Lines 121-130  (manuscript) |
| Data collection process | 9 | Specify the methods used to collect data from reports, including how many reviewers collected data from each report, whether they worked independently, any processes for obtaining or confirming data from study investigators, and if applicable, details of automation tools used in the process. | Lines 53-57  (supplementary material) |
| Data items | 10a | List and define all outcomes for which data were sought. Specify whether all results that were compatible with each outcome domain in each study were sought (e.g. for all measures, time points, analyses), and if not, the methods used to decide which results to collect. | Lines131-132  (manuscript) |
|  | 10b | List and define all other variables for which data were sought (e.g. participant and intervention characteristics, funding sources). Describe any assumptions made about any missing or unclear information. | Lines 59-108  (supplementary material) |
| Study risk of bias assessment | 11 | Specify the methods used to assess risk of bias in the included studies, including details of the tool(s) used, how many reviewers assessed each study and whether they worked independently, and if applicable, details of automation tools used in the process. | Lines143-149  (manuscript) |
| Effect measures | 12 | Specify for each outcome the effect measure(s) (e.g. risk ratio, mean difference) used in the synthesis or presentation of results. | Lines 134-137  (manuscript) |
| Synthesis methods | 13a | Describe the processes used to decide which studies were eligible for each synthesis (e.g. tabulating the study intervention characteristics and comparing against the planned groups for each synthesis (item #5)). | Lines 111-112  (supplementary material) |
|  | 13b | Describe any methods required to prepare the data for presentation or synthesis, such as handling of missing summary statistics, or data conversions. | Lines114-122  (supplementary material) |
|  | 13c | Describe any methods used to tabulate or visually display results of individual studies and syntheses. | Lines124-130  (supplementary material) |
|  | 13d | Describe any methods used to synthesize results and provide a rationale for the choice(s). If meta-analysis was performed, describe the model(s), method(s) to identify the presence and extent of statistical heterogeneity, and software package(s) used. | Lines131-135  (supplementary material) |
|  | 13e | Describe any methods used to explore possible causes of heterogeneity among study results (e.g. subgroup analysis, meta-regression). | Lines136-147  (supplementary material) |
|  | 13f | Describe any sensitivity analyses conducted to assess robustness of the synthesized results. | Lines139-149  (supplementary material) |
| Reporting bias assessment | 14 | Describe any methods used to assess risk of bias due to missing results in a synthesis (arising from reporting biases). | Lines156-160  (supplementary material) |
| Certainty assessment | 15 | Describe any methods used to assess certainty (or confidence) in the body of evidence for an outcome. | Lines150-156  (manuscript) |
| **RESULTS** | | |  |
| Study selection | 16a | Describe the results of the search and selection process, from the number of records identified in the search to the number of studies included in the review, ideally using a flow diagram. | Lines158-168  (manuscript) |
|  | 16b | Cite studies that might appear to meet the inclusion criteria, but which were excluded, and explain why they were excluded. | Lines158-161  (manuscript) |
| Study characteristics | 17 | Cite each included study and present its characteristics. | Lines168-178  (supplementary material) |
| Risk of bias in studies | 18 | Present assessments of risk of bias for each included study. | Lines249-261  (manuscript) |
| Results of individual studies | 19 | For all outcomes, present, for each study: (a) summary statistics for each group (where appropriate) and (b) an effect estimate and its precision (e.g. confidence/credible interval), ideally using structured tables or plots. | Lines174-179  (manuscript) |
| Results of syntheses | 20a | For each synthesis, briefly summarise the characteristics and risk of bias among contributing studies. | Lines192-202  (manuscript) |
|  | 20b | Present results of all statistical syntheses conducted. If meta-analysis was done, present for each the summary estimate and its precision (e.g. confidence/credible interval) and measures of statistical heterogeneity. If comparing groups, describe the direction of the effect. | Lines204-227  (manuscript) |
|  | 20c | Present results of all investigations of possible causes of heterogeneity among study results. | Lines191-202  (manuscript) |
|  | 20d | Present results of all sensitivity analyses conducted to assess the robustness of the synthesized results. | Lines180-190  (manuscript) |
| Reporting biases | 21 | Present assessments of risk of bias due to missing results (arising from reporting biases) for each synthesis assessed. | Lines260-271  (manuscript) |
| Certainty of evidence | 22 | Present assessments of certainty (or confidence) in the body of evidence for each outcome assessed. | Lines 272-280  (manuscript) |
| **DISCUSSION** | | |  |
| Discussion | 23a | Provide a general interpretation of the results in the context of other evidence. | Lines 283-368  (manuscript) |
|  | 23b | Discuss any limitations of the evidence included in the review. | Lines 369-378  (manuscript) |
|  | 23c | Discuss any limitations of the review processes used. | Lines 369-378  (manuscript) |
|  | 23d | Discuss implications of the results for practice, policy, and future research. | Lines 370-372  (manuscript) |
| **OTHER INFORMATION** | | |  |
| Registration and protocol | 24a | Provide registration information for the review, including register name and registration number, or state that the review was not registered. | Lines 418-420  (manuscript) |
|  | 24b | Indicate where the review protocol can be accessed, or state that a protocol was not prepared. | Lines 418-420  (manuscript) |
|  | 24c | Describe and explain any amendments to information provided at registration or in the protocol. | Lines 418-420  (manuscript) |
| Support | 25 | Describe sources of financial or non-financial support for the review, and the role of the funders or sponsors in the review. | Lines 423-425  (manuscript) |
| Competing interests | 26 | Declare any competing interests of review authors. | Lines 421-422  (manuscript) |
| Availability of data, code and other materials | 27 | Report which of the following are publicly available and where they can be found: template data collection forms; data extracted from included studies; data used for all analyses; analytic code; any other materials used in the review. | Lines 418-419  (manuscript) |

*From: Page MJ, McKenzie JE, Bossuyt PM, Boutron I, Hoffmann TC, Mulrow CD, et al. The PRISMA 2020 statement: an updated guideline for reporting systematic reviews. BMJ 2021;372:n71. doi: 10.1136/bmj.n71. This work is licensed under CC BY 4.0. To view a copy of this license, visit <https://creativecommons.org/licenses/by/4.0/>*

**References**

1. Treede, R. D., Rief, W., Barke, A., Aziz, Q., Bennett, M. I., Benoliel, R., ... & Wang, S. J. (2015). A classification of chronic pain for ICD-11. Pain, 156(6), 1003-1007.
2. Widiger, T. A., & Clark, L. A. (2000). Toward DSM—V and the classification of psychopathology. Psychological bulletin, 126(6), 946.
3. Wakefield, J. C. (1997). Diagnosing DSM-IV—Part I: DSM-IV and the concept of disorder. Behaviour research and therapy, 35(7), 633-649.
4. Okely, A. D. (1999). The relationship of participation in organised sports and games, participation in nonorganised physical activity, and cardiorespiratory endurance to fundamental motor skill ability among adolescents.
5. Howells, K., Sivaratnam, C., May, T., Lindor, E., McGillivray, J., & Rinehart, N. (2019). Efficacy of group-based organised physical activity participation for social outcomes in children with autism spectrum disorder: a systematic review and meta-analysis. Journal of Autism and Developmental Disorders, 49, 3290-3308.
6. Howells, K., Sivaratnam, C., May, T., Lindor, E., & Rinehart, N. (2019). A pilot acceptability study of an ‘AllPlay Pre-Learn’day program to facilitate participation in organised physical activity for children with disabilities. International Journal of Environmental Research and Public Health, 16(24), 5058.
7. Gavin, M. (2014). Fitness and your 2-3 year old. <https://kidshealth.org/en/parents/fitness-2-3.html>.
8. Bellamy, J., Broderick, C., Hardy, L. L., Simar, D., Puusepp‐Benazzouz, H., Ong, N., & Silove, N. (2020). Feasibility of a school‐based exercise intervention for children with intellectual disability to reduce cardio‐metabolic risk. Journal of intellectual disability research, 64(1), 7-17.
9. Khanjani, Z., Mosanezhad Jeddi, E., Hekmati, I., Khalilzade, S., Etemadi Nia, M., Andalib, M., & Ashrafian, P. (2015). Comparison of cognitive empathy, emotional empathy, and social functioning in different age groups. Australian Psychologist, 50(1), 80-85.
10. Cameron, N., & Bogin, B. (Eds.). (2012). Human growth and development. Academic Press.
11. Estrugo, Y., Bar Yehuda, S., & Bauminger‐Zviely, N. (2024). Motor, cognitive, and socio‐cognitive mechanisms explaining social skills in autism and typical development. Autism Research.
12. de Souza, M. S., Nobre, G. C., & Valentini, N. C. (2023). Effect of a motor skill-based intervention in the relationship of individual and contextual factors in children with and without Developmental Coordination Disorder from low-income families. Psychology of Sport and Exercise, 67, 102406.
13. Herold, F., Theobald, P., Gronwald, T., Kaushal, N., Zou, L., de Bruin, E. D., ... & Müller, N. G. (2024). Alexa, let's train now!—A systematic review and classification approach to digital and home-based physical training interventions aiming to support healthy cognitive aging. Journal of Sport and Health Science, 13(1), 30-46.
14. Bossavit, B., & Arnedillo-Sánchez, I. (2023). Motion-based technology to support motor skills screening in developing children: A scoping review. Computer Methods and Programs in Biomedicine, 107715.
15. Lubans, D. R., Lonsdale, C., Cohen, K., Eather, N., Beauchamp, M. R., Morgan, P. J., ... & Smith, J. J. (2017). Framework for the design and delivery of organized physical activity sessions for children and adolescents: rationale and description of the ‘SAAFE’teaching principles. International journal of behavioral nutrition and physical activity, 14, 1-11.
16. Toscano, C. V., Ferreira, J. P., Quinaud, R. T., Silva, K. M., Carvalho, H. M., & Gaspar, J. M. (2022). Exercise improves the social and behavioral skills of children and adolescent with autism spectrum disorders. Frontiers in psychiatry, 13, 1027799.
17. Borgi, M., Loliva, D., Cerino, S., Chiarotti, F., Venerosi, A., Bramini, M., ... & Cirulli, F. (2016). Effectiveness of a standardized equine-assisted therapy program for children with autism spectrum disorder. Journal of autism and developmental disorders, 46, 1-9.
18. Gabriels, R. L., Agnew, J. A., Holt, K. D., Shoffner, A., Zhaoxing, P., Ruzzano, S., ... & Mesibov, G. (2012). Pilot study measuring the effects of therapeutic horseback riding on school-age children and adolescents with autism spectrum disorders. Research in Autism Spectrum Disorders, 6(2), 578-588.
19. Sotoodeh, M. S., Arabameri, E., Panahibakhsh, M., Kheiroddin, F., Mirdoozandeh, H., & Ghanizadeh, A. (2017). Effectiveness of yoga training program on the severity of autism. Complementary Therapies in Clinical Practice, 28, 47-53.
20. Howells, K., Sivaratnam, C., Lindor, E., Hyde, C., McGillivray, J., Whitehouse, A., & Rinehart, N. (2020). Can participation in a community organized football program improve social, behavioural functioning and communication in children with autism spectrum disorder? A pilot study. Journal of autism and developmental disorders, 50, 3714-3727.
21. Lindor, E., Millard, O., Papadopoulos, N., Devenish, B. D., Bellows, S., Mantilla, A., ... & Rinehart, N. J. (2023). The feasibility and acceptability of AllPlay Dance for autistic children: A pilot randomised controlled trial. Research in Autism Spectrum Disorders, 109, 102271.
22. Caputo, G., Ippolito, G., Mazzotta, M., Sentenza, L., Muzio, M. R., Salzano, S., & Conson, M. (2018). Effectiveness of a multisystem aquatic therapy for children with autism spectrum disorders. Journal of autism and developmental disorders, 48, 1945-1956.
23. Teixeira-Machado, L., Arida, R. M., Ziebold, C., Barboza, A. B., Ribeiro, L., Teles, M. C., ... & de Jesus, J. M. (2022). A pilot randomized controlled clinical trial of dance practice for functionality in autistic children and adolescent with all levels of need support. Complementary Therapies in Clinical Practice, 49, 101650.
24. Movahedi, A., Bahrami, F., Marandi, S. M., & Abedi, A. (2013). Improvement in social dysfunction of children with autism spectrum disorder following long term Kata techniques training. Research in Autism Spectrum Disorders, 7(9), 1054-1061.
25. Gabriels, R. L., Pan, Z., Dechant, B., Agnew, J. A., Brim, N., & Mesibov, G. (2015). Randomized controlled trial of therapeutic horseback riding in children and adolescents with autism spectrum disorder. Journal of the American Academy of Child & Adolescent Psychiatry, 54(7), 541-549.
26. Li, Y., Feng, Y., Zhong, J., Zou, Z., Lan, W., Shen, Y., ... & Hou, X. (2023). The Effects of Physical Activity Interventions in Children with Autism Spectrum Disorder: a Systematic Review and Network Meta-analysis. Review Journal of Autism and Developmental Disorders, 1-15.
27. Li, L., Jia, S., Wang, P., Li, S., Wang, X., & Zhu, X. (2024). A network meta-analysis of the effect of physical exercise on core symptoms in patients with autism spectrum disorders. Frontiers in Neurology, 15, 1360434.
28. Oro, A. B., Navarro-Calvillo, M. E., & Esmer, C. (2014). Autistic Behavior Checklist (ABC) and its applications. Comprehensive guide to autism, 2787-2798.
29. Aksayli, N. D., Sala, G., & Gobet, F. (2019). The cognitive and academic benefits of Cogmed: A meta-analysis. Educational Research Review, 27, 229-243.
30. Viechtbauer, W. (2010). Conducting meta-analyses in R with the metafor package. Journal of statistical software, 36(3), 1-48.
31. Borenstein, M., Hedges, L. V., Higgins, J. P., & Rothstein, H. R. (2010). A basic introduction to fixed‐effect and random‐effects models for meta‐analysis. Research synthesis methods, 1(2), 97-111.
32. Morris, S. B. (2008). Estimating effect sizes from pretest-posttest-control group designs. Organizational research methods, 11(2), 364-386.
33. Schmidt, F. L., & Hunter, J. E. (2015). Methods of meta-analysis: Correcting error and bias in research findings (3rd ed.). Newbury Park, CA: Sage.
34. Assink, M., & Wibbelink, C. J. (2016). Fitting three-level meta-analytic models in R: A step-by-step tutorial. The Quantitative Methods for Psychology, 12(3), 154-174.
35. Cheung, M. W. L. (2014). Modeling dependent effect sizes with three-level meta-analyses: a structural equation modeling approach. Psychological methods, 19(2), 211.
36. Cheung, M. W. L. (2019). A guide to conducting a meta-analysis with non-independent effect sizes. Neuropsychology review, 29(4), 387-396.
37. Harrer, M., Cuijpers, P., Furukawa, T., & Ebert, D. (2021). Doing meta-analysis with R: A hands-on guide. Chapman and Hall/CRC.
38. Egger, M., Smith, G. D., Schneider, M., & Minder, C. (1997). Bias in meta-analysis detected by a simple, graphical test. bmj, 315(7109), 629-634.
39. Duval, S., & Tweedie, R. (2000). A nonparametric “trim and fill” method of accounting for publication bias in meta-analysis. Journal of the american statistical association, 95(449), 89-98.
40. Rodgers, M. A., & Pustejovsky, J. E. (2021). Evaluating meta-analytic methods to detect selective reporting in the presence of dependent effect sizes. Psychological methods, 26(2), 141.
41. Schindler, J., & Richter, T. (2023). Text generation benefits learning: A meta-analytic review. Educational Psychology Review, 35(2), 44.
42. Melby-Lervåg, M., Redick, T. S., & Hulme, C. (2016). Working memory training does not improve performance on measures of intelligence or other measures of “far transfer” evidence from a meta-analytic review. Perspectives on Psychological Science, 11(4), 512-534.
43. Cochran, W. G. (1954). The combination of estimates from different experiments. Biometrics, 10(1), 101-129.
44. Díaz-Quesada, G., Gálvez-Calabria, M. D. L. Á., Connor, J. D., & Torres-Luque, G. (2022). When are children most physically active? An analysis of preschool age children’s physical activity levels. Children, 9(7), 1015.
45. Nagovitsyn, R. S., Tutolmin, A. V., Maksimov, Y. G., Dimova, I. A., Karoyan, A. A., Skryabina, D. Y., & Volkov, S. A. (2019). Motivation for physical activity of people of different ages. Gazzetta Medica Italiana Archivio per le Scienze Mediche, 178(10), 799-806.
46. Liutsko, L., Leonov, S., Pashenko, A., & Polikanova, I. (2024). Is Frequency of Practice of Different Types of Physical Activity Associated with Health and a Healthy Lifestyle at Different Ages?. European Journal of Investigation in Health, Psychology and Education, 14(1), 256-271.
47. Harrell, F. E. (2001). Regression modeling strategies: with applications to linear models, logistic regression, and survival analysis (Vol. 608). New York: springer.
48. Wiium, N., & Säfvenbom, R. (2019). Participation in organized sports and self-organized physical activity: Associations with developmental factors. International journal of environmental research and public health, 16(4), 585.
49. McNeill, J., Howard, S. J., Vella, S. A., Santos, R., & Cliff, D. P. (2018). Physical activity and modified organized sport among preschool children: Associations with cognitive and psychosocial health. Mental Health and Physical Activity, 15, 45-52.
50. Mosoi, A. A., Beckmann, J., Mirifar, A., Martinent, G., & Balint, L. (2020). Influence of organized vs non organized physical activity on school adaptation behavior. Frontiers in Psychology, 11, 550952.
